# Domino Strategies in Heterocycle Synthesis: Advancing from Organocatalysis to Photo-/Organo-Autocatalysis

**DOI:** 10.1021/acscentsci.6c00561

**Published:** 2026-07-13

**Authors:** Abdulaziz A. Al-Romema, Felix Heckmann, Svetlana B. Tsogoeva

**Affiliations:** Department of Chemistry and Pharmacy, Friedrich-Alexander-Universität Erlangen-Nürnberg, 91058 Erlangen, Germany

## Abstract

The increasing structural complexity of heterocycles in pharmaceuticals, agrochemicals, and functional materials continues to challenge synthetic design, particularly when step economy, scalability, and sustainability are considered simultaneously. Domino reactions, in which multiple bond-forming events proceed sequentially in a single operation, offer an inherently concise strategy to transform simple building blocks into densely functionalized architectures while minimizing purification steps and waste. In this *Outlook*, we examine the evolution of domino heterocycle synthesis from classical organocatalysis to photo- and organo-autocatalytic systems, highlighting how metal-free activation, visible-light processes, and *in situ*-generated catalytic species expand reactivity while improving catalyst, energy, and atom economy. We discuss key advances in domino reactions toward chiral heterocycles, heteroaromatic construction, total synthesis, and self-catalyzed photochemical processes, emphasizing challenges such as catalyst loading, robustness, photon efficiency, and reaction scalability. The future development of domino synthesis will rely on combining new mechanistic ideas with sustainability and rational reaction design. By focusing on the deliberate development of efficient, low-input organo*-* and photo/organo-autocatalyzed domino reactions, we aim to spur progress toward streamlined and industrially practical methods for heterocycle synthesis.

## Graphical abstract

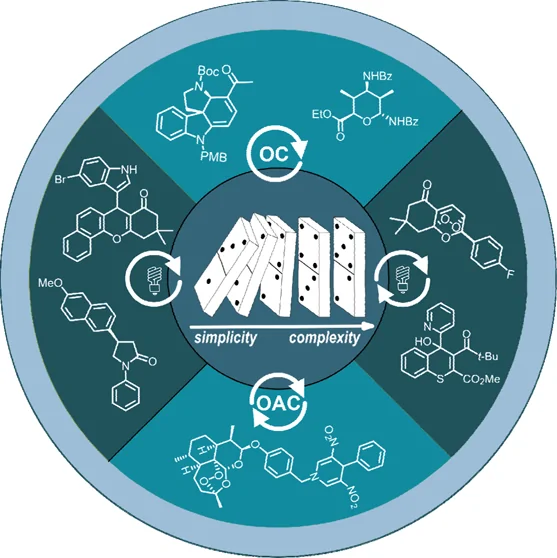

## 1 Introduction

Simplicity in organic synthesis is achieved when molecular complexity can be constructed efficiently, selectively, and with minimal resource expenditure in a single operation. In recent decades, the synthesis of complex heterocyclic frameworks—central motifs in pharmaceuticals, natural products, agrochemicals, ligands, and functional materials—has matured into a highly sophisticated field of research.^1–8^ Nevertheless, many established routes still rely on extended protecting-group manipulations, discrete isolation of intermediates, and resource-intensive purification steps that arise from traditional stepwise or “Stop-and-Go” strategies (Figure 1A). Such approaches, although reliable, remain distant from the ideals of modern synthesis, which emphasize atom economy, step and pot efficiency, reduced solvent use, and, ideally, protecting-group-free transformations.^9–11^

**Figure 1:**
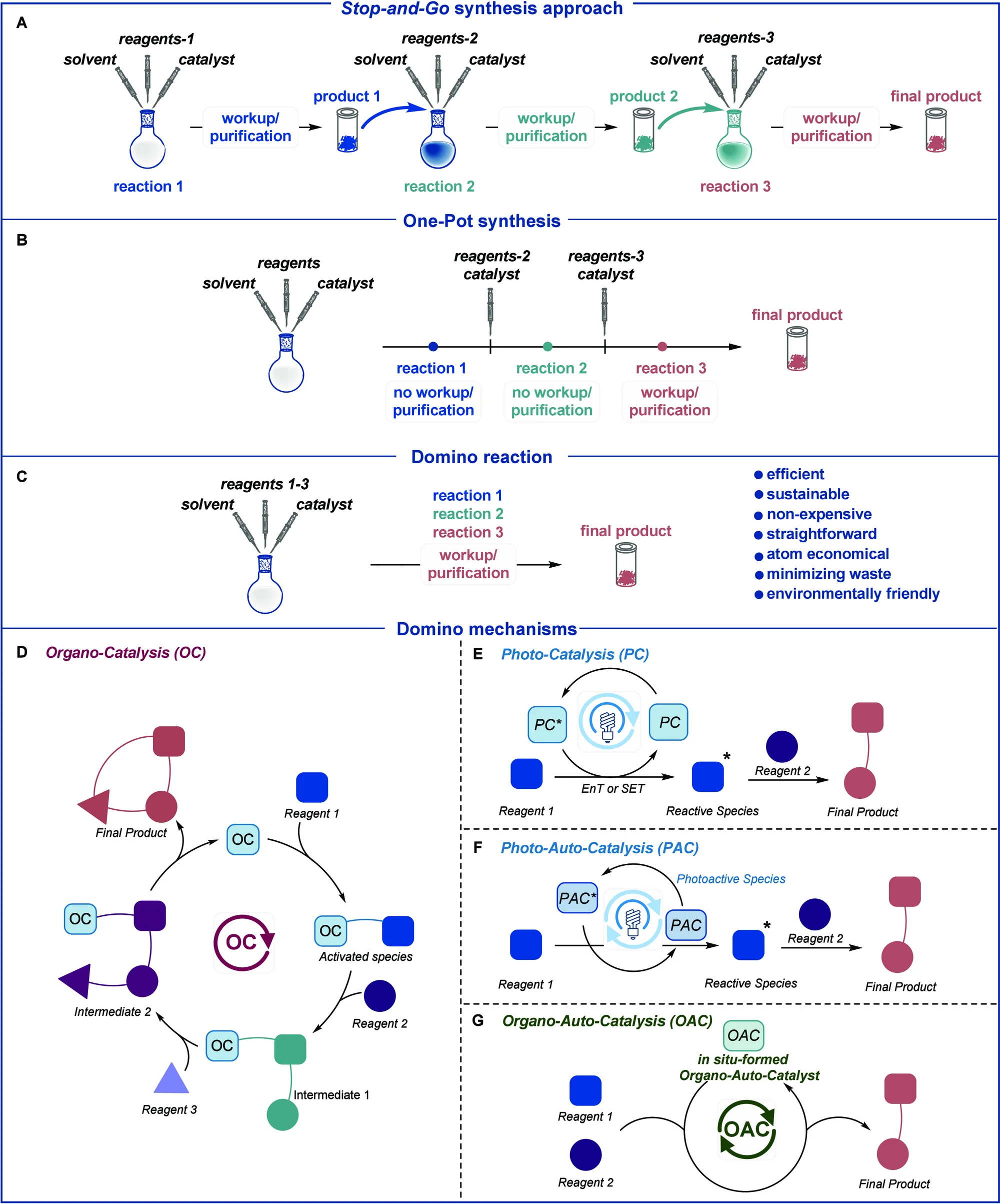
Strategies for heterocycle synthesis: A) Classical Stop-and-Go approach; B) One-pot approach; C) Domino reaction principle; D) Organocatalyzed domino reaction; E) Photocatalyzed domino; F) Photoautocatalyzed domino; G) Organo-autocatalyzed domino.

As organic chemistry increasingly aligns with the principles of sustainable development, efficiency and waste reduction have become not merely a practical consideration but a defining design criterion.^12,13^ Transformations are now commonly evaluated using quantitative metrics such as atom economy and process mass intensity, while lifecycle thinking continues to shape both academic research and industrial practice.^14^ In this context, a reaction design that minimizes operational steps and maximizes bond-forming efficiency offers intrinsic advantages.

One-pot processes^15–20^ and domino reactions^21–26^ have therefore emerged as powerful strategic concepts for constructing molecular complexity from simple starting materials in a streamlined and resource-conscious manner. Although often discussed together,^16,27^ one-pot and domino reactions embody distinct operational philosophies. In one-pot processes, individual transformations are performed sequentially within a single reaction vessel; upon completion of each step, additional reagents or catalysts are introduced to trigger the subsequent event, culminating in product formation after a single final isolation (Figure 1B). Domino reactions, in contrast, are initiated in the presence of all necessary components—reagents, catalysts, and solvent—such that once the first bond-forming event occurs, subsequent transformations proceed autonomously without further external intervention (Figure 1C). Each elementary step directly triggers the next, enabling the uninterrupted assembly of structurally intricate architectures through a self-propagating domino process. By reducing workup operations and avoiding intermediate purifications, these methodologies significantly reduce solvent consumption, material loss, and energy demand, underscoring their growing importance as a superior tool for organic synthesis.^15–29^

While domino reactions had been known in racemic contexts for decades, the first example of an asymmetric domino reaction was reported in 1998 by Terashima and co-workers.^30^ The synthesis of the naturally occurring acetylcholinesterase inhibitor (−)-Huperzine A was accomplished through a Cinchona alkaloid–promoted asymmetric two-step Michael addition/aldol reaction domino transformation between a β-keto ester and methacrolein (Scheme 1). This domino process afforded the key intermediate in 45% yield, which was subsequently elaborated to complete the total synthesis of (−)-Huperzine A with 64% ee. This work pioneered the idea that a single chiral organic compound could promote multiple bond-forming events in a single operation with enantioselective control, foreshadowing the later explosion of asymmetric organocatalytic cascade methodologies. Subsequent developments in the early 2000s, especially in asymmetric organocatalysis, built directly on this conceptual advance to enable highly enantioselective reactions with broad synthetic utility. Asymmetric organocatalysis, recognized with the 2021 *Nobel Prize* awarded to Benjamin List and David MacMillan for its development,^31,32^ utilizes small organic molecules as chiral catalysts, such as alkaloid derivatives,^33–35^ amino acids,^36^ short peptides,^37^ (thio)­ureas,^38–42^ and chiral Brønsted acids.^43–49^

**Scheme 1:**
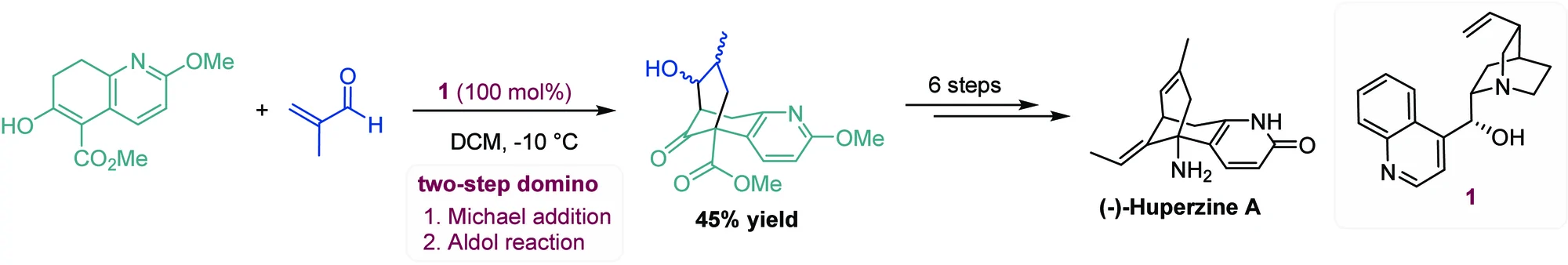
First Example of an Enantioselective Domino Reaction: Total Synthesis of the Acetylcholinesterase Inhibitor (−)-Huperzine A

Since its renaissance in the early 2000s by MacMillan, List, and Barbas,^46,50–53^ organocatalysis has demonstrated remarkable versatility, significantly advancing the field of asymmetric organic synthesis.^54–65^

Over recent decades, a substantial body of research has demonstrated the synthetic power of organocatalyzed domino reactions in the construction of heterocyclic scaffolds.^66–68^ This *Outlook* examines the conceptual principles and emerging directions of domino strategies in heterocycle synthesis, with a focus on *organo-*, *photo-*, and *autocatalytic* systems (Figures 1D-G). By integrating metal-free activation modes, visible-light-driven processes, and self-propagating catalytic networks, these approaches enable the construction of diverse and densely functionalized heterocyclic frameworks with high atom and step economy. Beyond showcasing the synthetic elegance of domino reactions, this *Outlook* critically evaluates how domino strategies can address contemporary challenges of catalyst economy, robustness, reaction scalability, and potential industrial applications. Special emphasis is placed on the transition from conventional organocatalysis (Figure 1D) and photo-catalysis (Figure 1E) to photo-autocatalytic (Figure 1F) and organo-autocatalytic (Figure 1G) reactions, where substrates, *in situ*-generated intermediates, or products actively participate in sustaining the catalytic cycle, thereby reducing external inputs and improving sustainability metrics. Rather than cataloging reactions exhaustively, this Outlook highlights representative examples and advances that illustrate how domino reactions can reconcile molecular complexity with operational simplicity. Key case studies from organocatalyzed non-enantioselective and enantioselective heterocycle synthesis, heteroaromatic construction, total synthesis of bioactive compounds, and photo-auto- and organo-autocatalyzed reactions are discussed to demonstrate the new directions of the field. By framing heterocycle construction through the lens of single-operation synthesis, we aim to highlight the unifying principles that connect organo-catalysis, photocatalysis, and photo-/organo-autocatalysis and to inspire further developments toward increasingly streamlined, waste-reducing, energy-conscious, and sustainable synthetic strategies capable of meeting future environmental and industrial demands.

Beyond showcasing the synthetic elegance of domino reactions, this Outlook critically evaluates how domino strategies can address contemporary challenges of catalyst economy, robustness, reaction scalability, and potential industrial applications.

## 2 Non-Enantioselective Synthesis of Heterocycles

The construction of chiral heterocycles without the use of enantiopure catalysts represents a conceptually distinct yet strategically useful approach to complexity generation.

In such processes, multiple stereogenic centers are established in a single operation, often with high levels of diastereocontrol, while the absolute configuration remains uncontrolled. From the perspective of synthetic efficiency, these transformations are highly attractive: they avoid the preparation of chiral catalysts or auxiliaries, simplify reaction design, and frequently deliver functionalized scaffolds suitable for downstream diversification and chiral resolution. The following sections highlight representative advances in the non-enantioselective domino synthesis of mono-, multi-, and bridged heterocyclic frameworks.

### 2.1 Monocyclic Heterocycles

These structures are key motifs in medicinal and synthetic organic chemistry, valued for their three-dimensionality, drug-like properties, and prevalence in bioactive molecules. Recent reviews have summarized advances in their preparation, including direct alkene difunctionalization strategies and modular approaches to saturated N-heterocycles from common building blocks.^7^ Among notable organocatalytic contributions, Lupton and co-workers reported an N-heterocyclic carbene (NHC)-catalyzed domino strategy for the synthesis of dihydropyranones (Scheme 2A).^69^ In this transformation, α,β-unsaturated enol esters or acid fluorides react with TMS enol ethers *via* Lewis base–promoted fragmentation to generate reactive acyl azolium intermediates. A three-step domino sequence—Michael addition/proton transfer/cyclizing acylation—furnishes pyranones in yields of up to 76%, with efficient catalyst regeneration. This work demonstrates how transient acyl azolium species can be harnessed to construct oxygen heterocycles through domino reactivity. Manolikakes and co-workers developed a highly stereoselective domino protocol for the synthesis of fully substituted tetrahydropyrans (Scheme 2B).^70^ The process involves a 2-fold addition of enamides to aldehydes, followed by cyclization, proceeding through a 1-carbonyl-ene-type/aza-ene-type/cyclization sequence. Three new σ-bonds and five contiguous stereogenic centers are formed in a single operation, often delivering a single diastereomer out of 16 possible isomers. Notably, selective use of *E*- or *Z*-configured enamides enables controlled access to distinct stereoisomeric products, underlining the power of substrate-controlled stereodifferentiation in achiral catalytic systems.

**Scheme 2:**
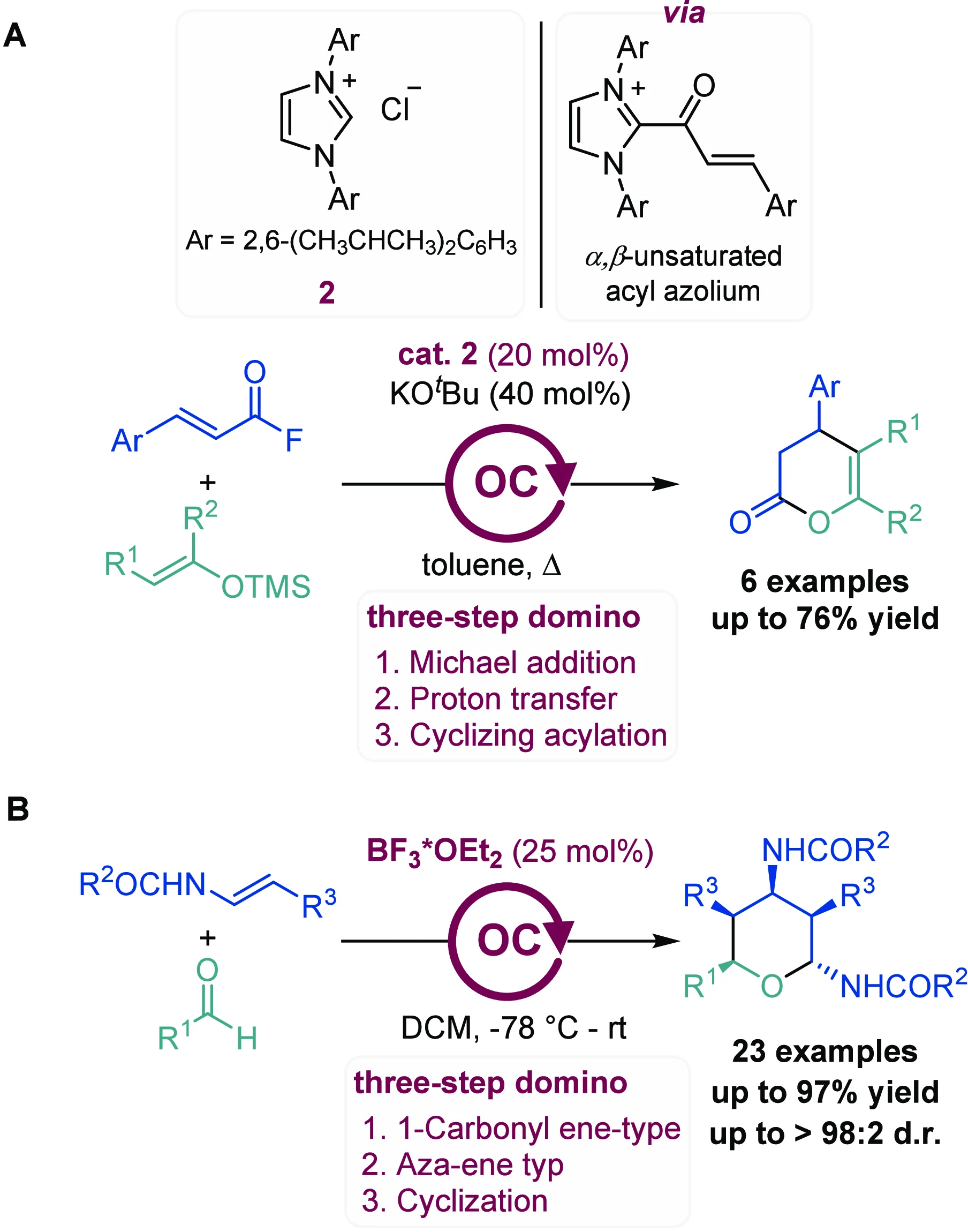
Non-Enantioselective Synthesis of Monocyclic Heterocycles: A) Dihydropyranones; B) Tetrahydropyrans

### 2.2 Multicyclic Heterocycles

These compounds, including fused and annulated systems, are prominent structural motifs in natural products and bioactive molecules. Their rigid architectures often enhance biological activity and metabolic stability. While transition-metal catalysis has long dominated their synthesis,^8^ organocatalytic domino strategies have emerged as complementary, more cost-efficient, and operationally simple alternatives.^6^

In this context, Huang, Chen, and co-workers reported a phosphine-catalyzed domino reaction enabling access to highly substituted *trans*-2,3-dihydrobenzofurans with excellent diastereoselectivity (Scheme 3A).^71^ Salicyl *N*-thiophosphinyl imines and allylic carbonates, acting as 1,1-dipolar synthons, undergo a four-step domino reaction comprising addition–elimination/nucleophilic addition/isomerization/intramolecular annulation. The method affords dihydrobenzofurans in yields of up to 99%, illustrating the efficiency of phosphine catalysis in controlling multistep sequences. Takizawa, Sasai, and co-workers developed a Lewis base–catalyzed domino reaction between cyclohexadienones and alkynyl esters to synthesize hydroindole-2-carboxylates and hydrobenzofuran-2-carboxylates (Scheme 3B).^72^ The four-step sequence—Michael addition/umpolung Michael addition/Michael addition/proton transfer—provides up to 20 derivatives in yields of up to 89%. This example highlights the strategic use of polarity inversion (umpolung) within a domino framework to access complex bicyclic systems (Figure 2). Along this line, Tong and co-workers disclosed a phosphine-catalyzed synthesis of cyclopenta­[a]­pyrrolizine derivatives from 2-acyl-3-(2-pyrrole)-acrylonitriles and β′-acetoxy allenoates (Scheme 3C).^73^ The three-step domino process—addition–elimination/aza-Michael addition/intramolecular [3 + 2] cycloaddition—furnishes products in yields of up to 95%. Complementarily, Piersanti and co-workers described a phosphoric acid–catalyzed approach to 3-NHAc-naphtho- and benzofuranones from 2-acetamidoacrylates and naphthols or phenols (Scheme 3D).^74^ Proceeding *via* dual activation through protonation and hydrogen bonding, the two-step domino sequence—aza-Friedel–Crafts reaction/intramolecular cyclization—delivers products in up to 67% yield.

**Scheme 3:**
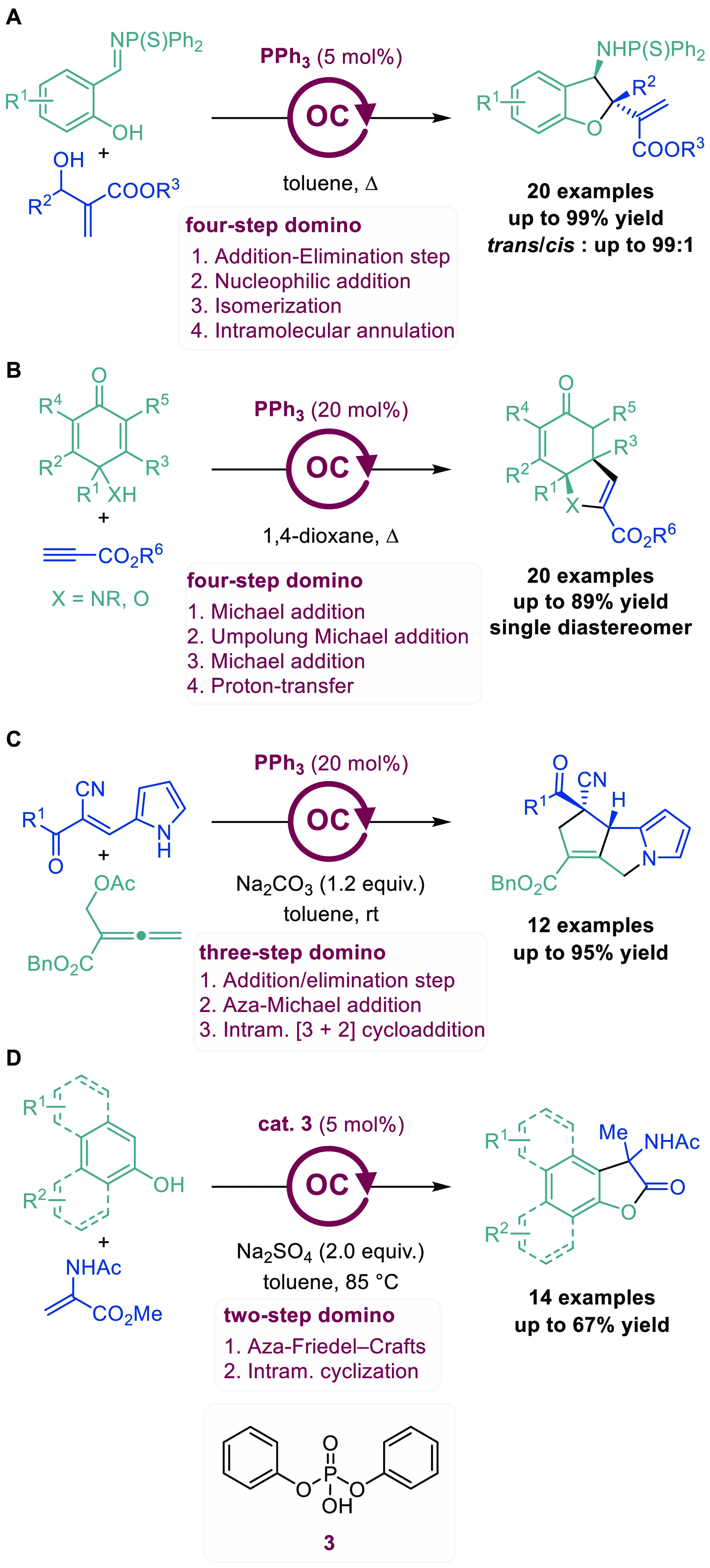
Non-Enantioselective Synthesis of Multicyclic Heterocycles: A) *trans*-2,3-Dihydrobenzofurans; B) Hydroindole-2-carboxylates and Hydrobenzofuran-2-carboxylates; C) Cyclopenta­[a]­pyrrolizines; D) Naphtho- and Benzofuranones

**Figure 2:**
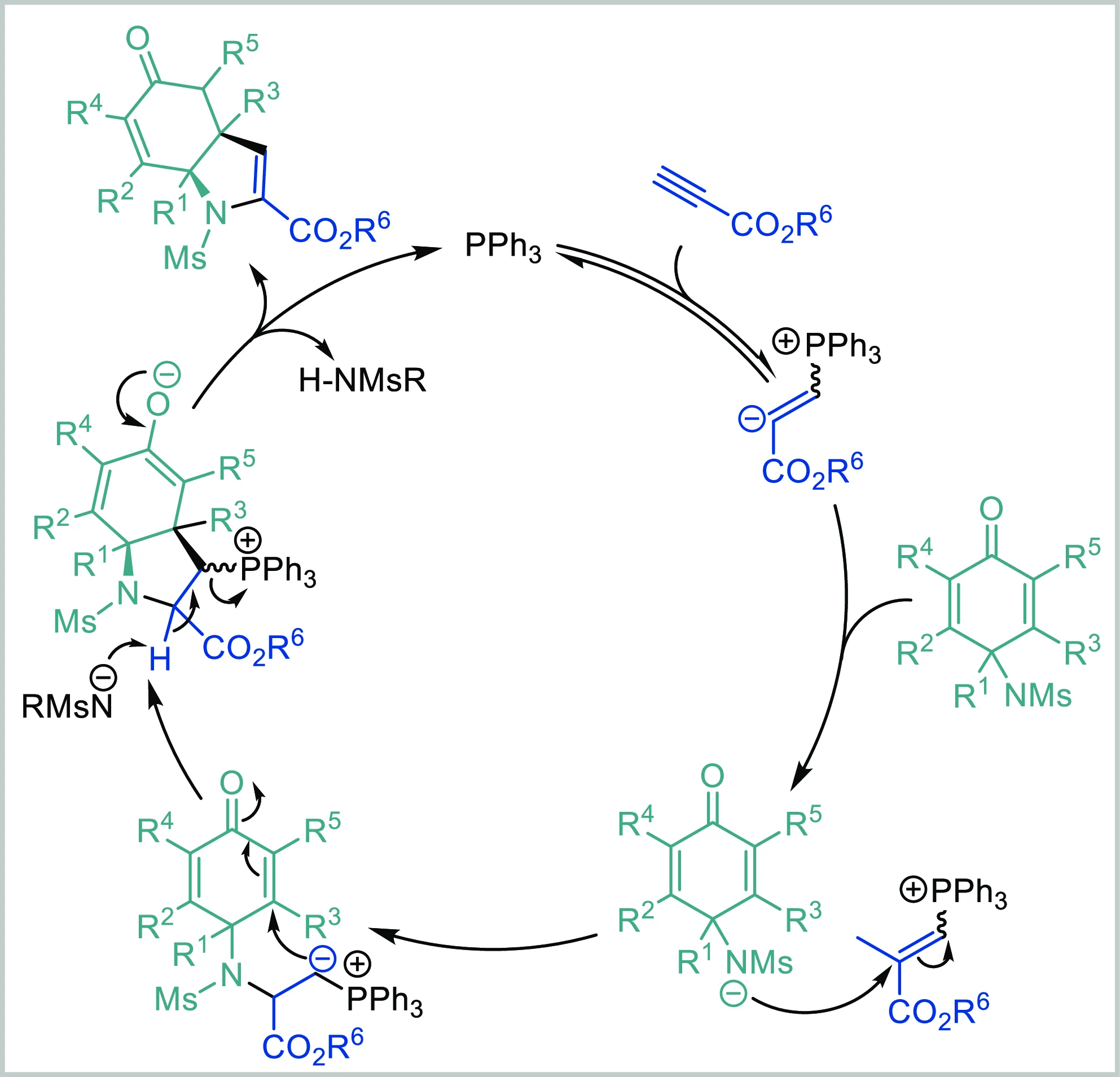
Plausible mechanism for the domino transformation presented in Scheme 3B

### 2.3 Bridged Heterocycles

These structures represent complex architectures frequently encountered in natural products and bioactive compounds. Their synthesis poses significant challenges due to ring strain and the requirement for precise stereochemical control.

Domino strategies have proven particularly well-suited to their rapid assembly. Along these lines, Tong and co-workers developed a phosphine-catalyzed domino reaction between β′-acetoxy allenoates and 2-acyl-3-methyl-acrylonitriles to access 2-oxabicyclo[3.3.1]­nonane derivatives (Scheme 4A).^73^ In this five-step domino process—addition–elimination step/nucleophilic addition/intramolecular Michael addition/H-shift/S_N_2-type reaction—the β′-carbon of the allenoate serves as an electrophilic center, while the β′- and γ-carbons function as a 1,4-dipole (Figure 3). Up to 15 derivatives were synthesized in yields of up to 87%, underscoring the efficiency of sequential bond construction within a single operational setting.

**Scheme 4:**
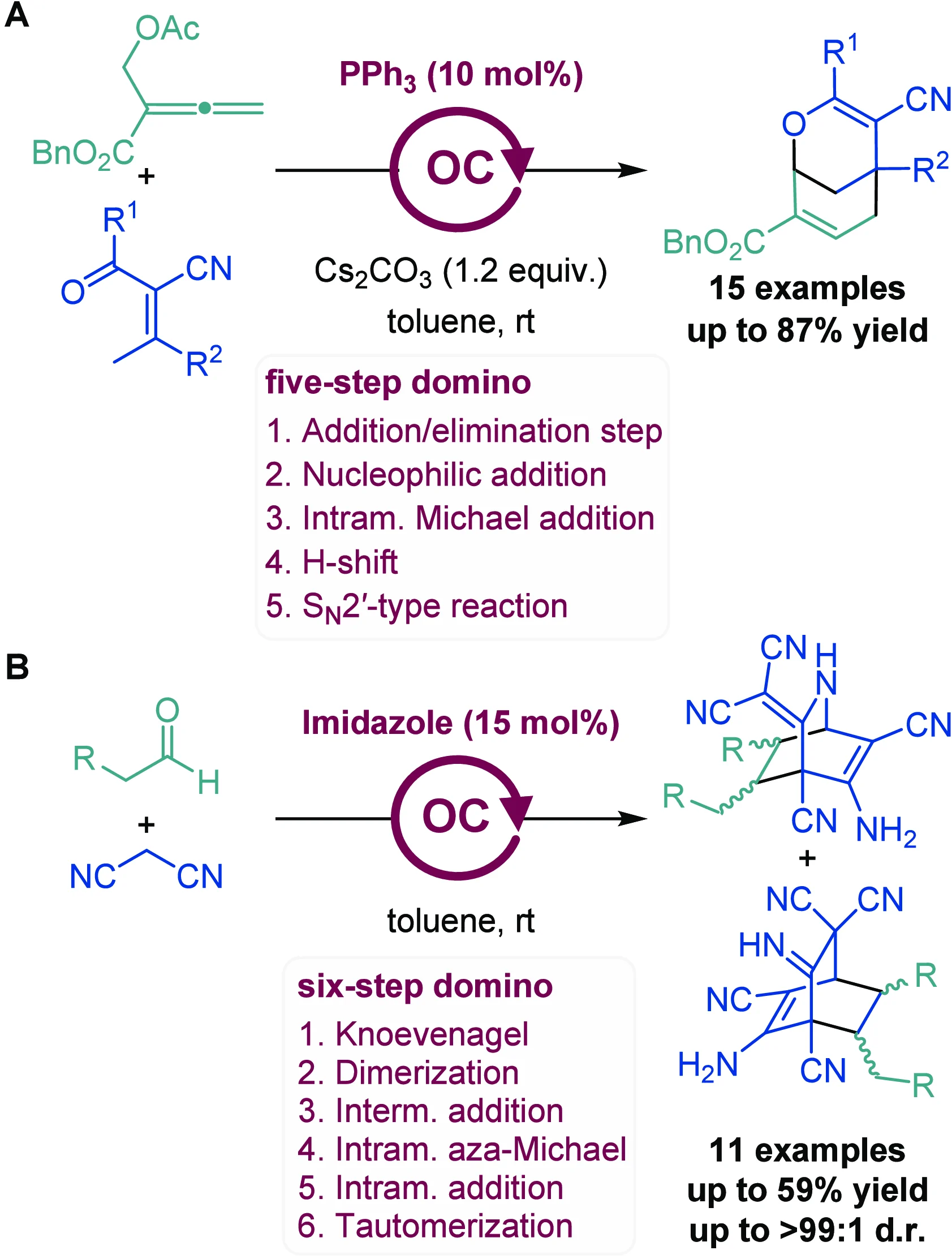
Non-Enantioselective Synthesis of Bridged Heterocycles: A) 2-Oxabicyclo[3.3.1]­nonanes; B) Aza- and Carbo-bicycles

**Figure 3:**
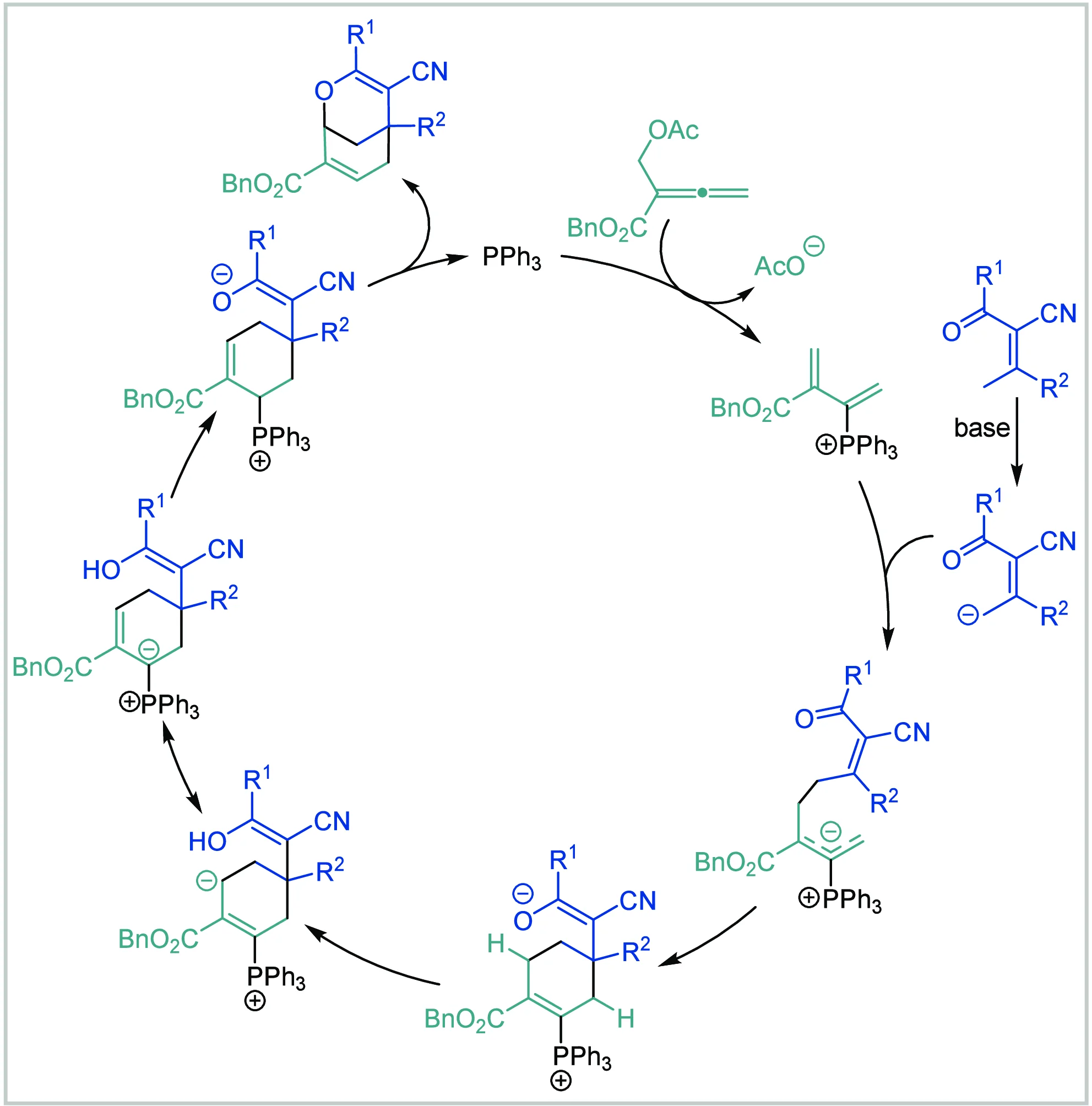
Proposed mechanisms for the domino reaction depicted in Scheme 4A.

The Tsogoeva group reported an imidazole-catalyzed domino reaction between malononitrile and substituted aldehydes, enabling access to complex aza- and carbo-bicycles (Scheme 4B).^75^ The six-step sequence—Knoevenagel condensation/dimerization/intermolecular addition/intramolecular aza-Michael reaction/intramolecular addition/tautomerization—provides 11 derivatives in up to 59% yield. This transformation exemplifies how simple organocatalysts can control intricate reaction networks to generate bridged frameworks from readily available starting materials.

The examples discussed in this chapter illustrate that achiral catalytic systems can deliver highly complex, stereochemically rich heterocycles through carefully designed domino sequences. While non-enantioselective approaches offer advantages such as operational simplicity, easy access to catalysts, and rapid scaffold construction, enantioselective catalysis remains essential for directly obtaining single enantiomers.

## 3 Enantioselective Organocatalyzed Synthesis of Heterocycles

The enantioselective construction of chiral heterocycles represents a central objective in modern synthetic chemistry, particularly in view of the profound influence of absolute configuration on biological activity and pharmacological properties.^76–78^ Domino strategies, when combined with asymmetric organocatalysts, enable the simultaneous formation of multiple stereogenic centers with high levels of enantiocontrol in a single operational step. Asymmetric organocatalyzed domino reactions combine step economy with control over stereochemistry, making them a powerful tool for building complex chiral molecules.^79^ Despite recent impressive advances, major challenges remain, including substrate generality, chiral organocatalyst cost, and the compatibility of highly reactive intermediates within complex domino manifolds. Furthermore, the translation of optimized academic examples to industrially viable processes continues to represent a significant hurdle. The following subsections highlight representative examples in which carefully designed chiral organocatalysts facilitate domino processes to access enantioenriched heterocyclic compounds with excellent levels of diastereo- and enantioselectivity. The question is not only how high the enantiomeric excess can be driven, but how broadly applicable, and sustainable such asymmetric domino strategies can ultimately become.

### 3.1 Monocyclic Heterocycles

Among the notable contributions exemplifying recent advances is the work by Ye and co-workers who demonstrated the enantioselective synthesis of challenging β-lactams. The reported method employs chiral NHC precatalysts to activate ketenes, followed by reaction with a variety of *N-tert*-butoxycarbonyl arylamines (Scheme 5A).^80^ The reaction proceeds *via* a two-step nucleophilic addition/intramolecular cyclization domino process (Figure 4) affording *cis-β*-lactams in good yields and in up to 99% ee. While the method delivers excellent enantioselectivity, the reliance on preformed ketenes and tailored NHC precatalysts may limit large-scale adoption, highlighting the ongoing need for more readily accessible catalytic systems and milder activation modes.

**Scheme 5:**
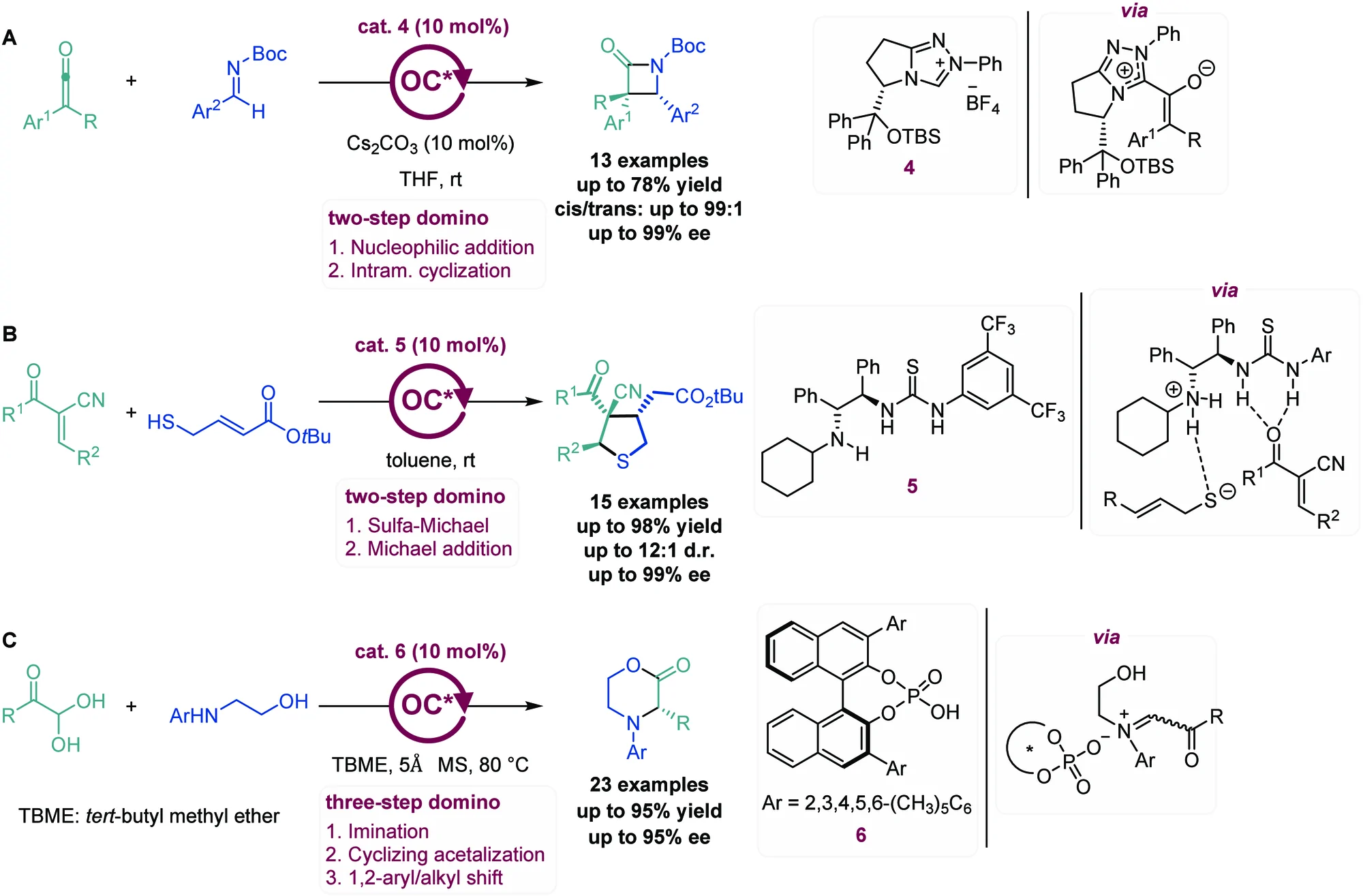
Enantioselective Synthesis of Monocyclic Heterocycles: A) *β*-Lactams; B) Tetrahydrothiophenes; C) Morpholinones

**Figure 4:**
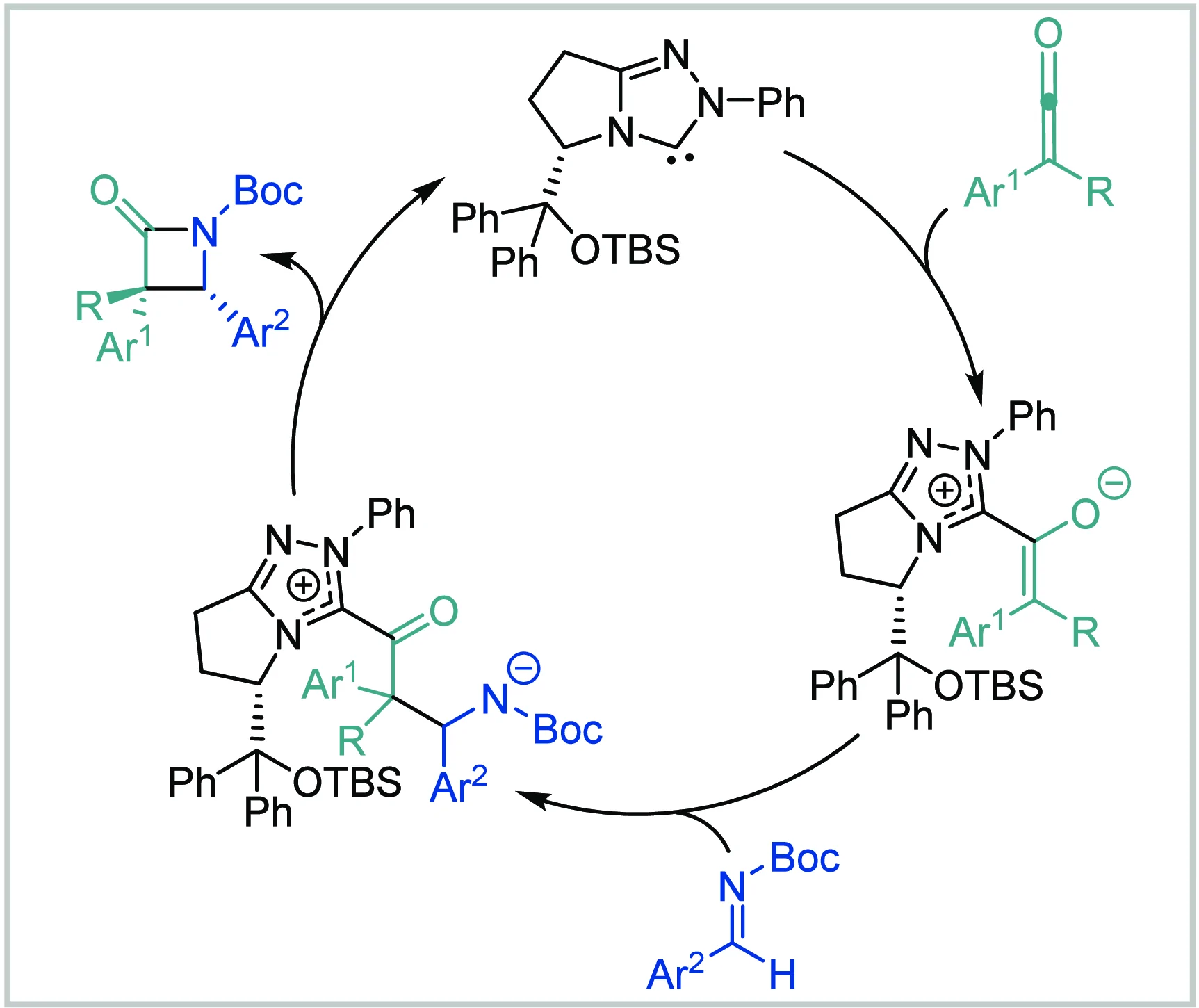
Plausible reaction mechanism for the domino transformations presented in Scheme 5A.

Highly functionalized tetrahydrothiophenes with three contiguous stereocenters were synthesized by the group of Lattanzi using *trans-α*-cyano-α,β-unsaturated ketones, *trans-tert*-butyl 4-mercapto-2-butenoate, and a bifunctional chiral amine-thiourea organocatalyst (Scheme 5B).^81^ The transformation proceeds *via* a two-step sulfa-Michael/Michael domino process, delivering 15 examples with high diastereoselectivity and enantioselectivity (up to 99% ee) and excellent yields of up to 98%. The formation of multiple contiguous stereocenters in a single operation illustrates the power of bifunctional catalysis. Using a chiral phosphoric acid Zhu and the team established an enantioselective synthesis to access morpholinone substrates from aryl/alkylglyoxals and 2-(arylamino)­ethan-1-ols (Scheme 5C).^82,83^

A total of 23 derivatives were synthesized in excellent yields and up to 95% ee through a three-step domino reaction comprised of imination/cyclizing azetalization/1,2-aryl-alkyl shift.

Such domino transformations highlight the synthetic creativity enabled by asymmetric organocatalysis, yet mechanistic complexity and potential competing pathways underscore the delicate balance required for reliable domino reaction design.

### 3.2 Bicyclic Heterocycles

A highly diastereoselective and enantioselective synthetic route toward 1,3-disubstituted isoindolines was reported by the Sasai group utilizing a chiral bifunctional (acid–base) organocatalyst for the reaction of electron-deficient alkenes with *N*-tosylimines (Scheme 6A).^8^ ^3^

**Scheme 6:**
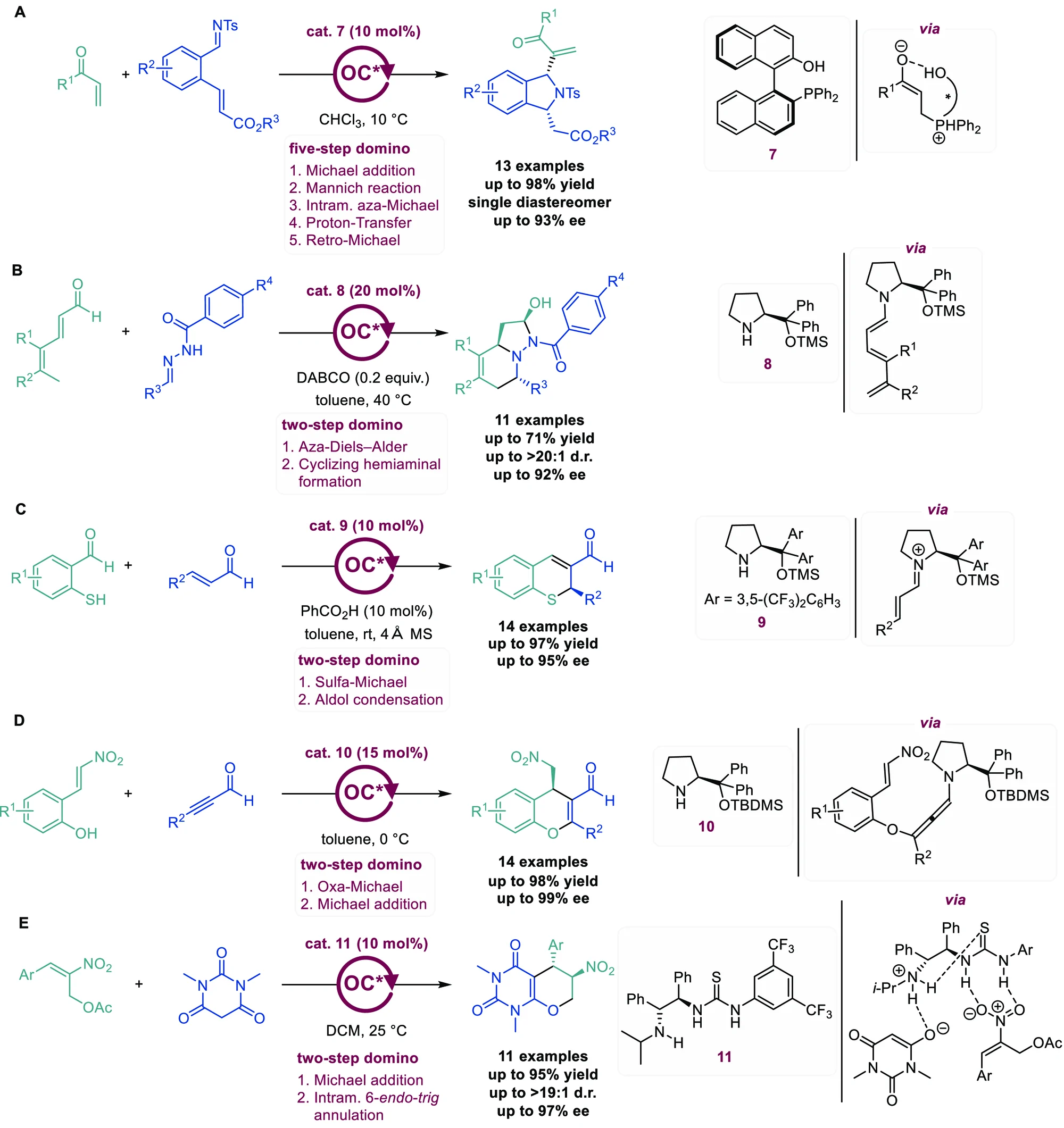
Enantioselective Synthesis of Bicyclic Heterocycles: A) Isoindolines; B) Bicyclic Azaheterocycles; C) Thiochromenes; D) 4*H*-Chromenes; E) Barbiturate-Tetrahydropyran Fused Heterocycles

The transformation describes a five-step Michael addition/Mannich reaction/intramolecular aza-Michael/proton transfer/retro-Michael domino reaction yielding the final products in good yields and in up to 93% ee. The control of five consecutive elementary steps within a single catalytic cycle demonstrates the sophistication of modern domino design; nevertheless, increasing reaction robustness and reducing sensitivity to electronic effects remain ongoing challenges.

An interesting approach for the *in situ* generation of activated trienamines was developed by the group of Jørgensen (Scheme 6B).^84^ The organocatalyzed activation of 2,4-dienals forms the reactive trienamine species which undergo a two-step aza-Diels–Alder/cyclizing hemiaminal formation domino process to yield the desired azaheterocycles in moderate to good yields (up to 71%) in up to >20:1 d.r. and 92% ee.

The application of the method was further demonstrated by transforming the obtained azaheterocycles into tetrahydropyridine motif found in palustrine derivatives such as (−)-methyl palustramate or (+)-cannabisativine.The synthetic utility toward complex alkaloid frameworks is notable; and further improvements in yields and reducing catalyst loading would enhance the practicality of such activation modes.

For the asymmetric synthesis of thiochromenes, Wang and co-workers reported an organocatalyzed two-step sulfa-Michael aldol condensation domino reaction (Scheme 6C).^85^ The activation of α,β-unsaturated aldehydes with chiral organocatalyst followed by a reaction with 2-mercaptobenzaldehyde provided the final products in high yields and up to 95% ee. Subsequently, an asymmetric synthesis of functionalized 4*H*-chromenes was developed by the same group. The novelty of this work was the unprecedented iminium–allenamine cascade activation resulting from the utilization of alkynals as starting materials (Scheme 6D).^86^ This undergoes a two-step oxa-Michael/Michael domino reaction with 2-(*E*)-(2-nitrovinyl)-phenols affording the products in high yields and up to 99% ee.

Innovative activation concepts such as iminium–allenamine cascades (Figure 5) expand the conceptual toolbox of asymmetric catalysis, yet they also raise questions concerning generality and long-term operational simplicity. The synthesis of barbiturate-tetrahydropyran fused heterocycles utilizing a bifunctional (amine-thiourea) organocatalyst was presented by Li, Chen and co-workers (Scheme 6E).^87^ A two-step Michael addition/intramolecular 6-*endo-trig* annulation domino reaction between *N*, *N*′-dimethylbarbituric acid and Morita-Baylis-Hillman (MBH) acetates of nitroalkenes afforded the fused structures in good yields and up to >19:1 d.r. and 97% ee. Future progress will likely depend on integrating such highly selective systems with scalable reaction engineering and reduced catalyst loadings.

**Figure 5:**
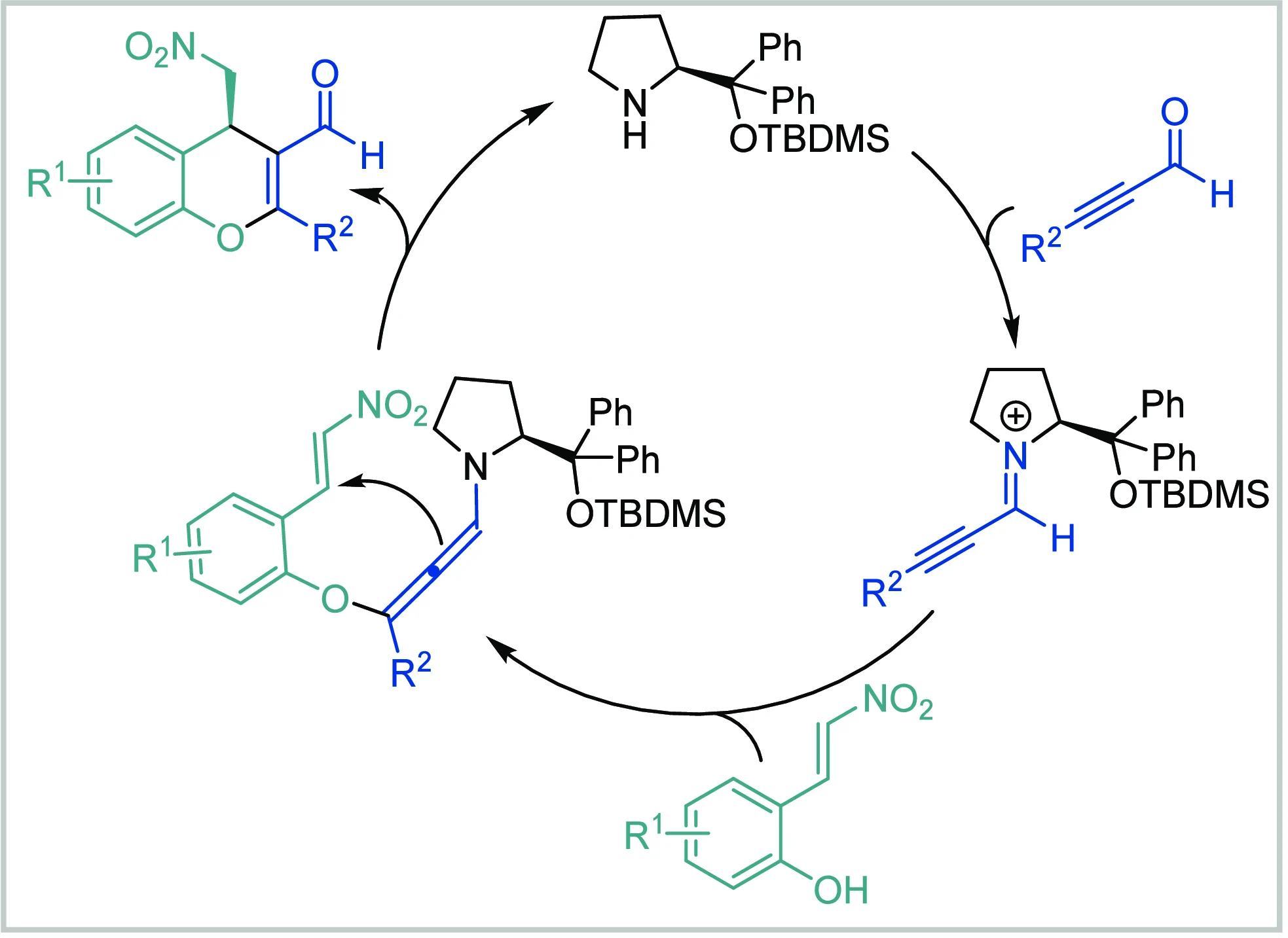
Proposed mechanisms for the domino reaction depicted in Scheme 6D.

### 3.3 Multicyclic Heterocycles

Ohsawa and his team reported the first catalytic asymmetric reaction of 3,4-dihydro-β-carboline and 3-buten-2-one in combination with chiral catalyst (*S*)-Proline to yield 3,4,6,7,12,12b-hexahydro-1*H*-indolo­[2,3-*a*]­quinolizin-2-one (Scheme 7A).^88^ The target compound was obtained *via* a two-step Mannich reaction/aza-Michael addition domino sequence, providing the product in good yield and 92% ee. The obtained scaffold represents a valuable structure for the total synthesis of indole alkaloids. Such examples illustrate how asymmetric domino catalysis can rapidly access alkaloid-like frameworks. However, expanding these strategies to more densely functionalized substrates remains an important frontier.

**Scheme 7:**
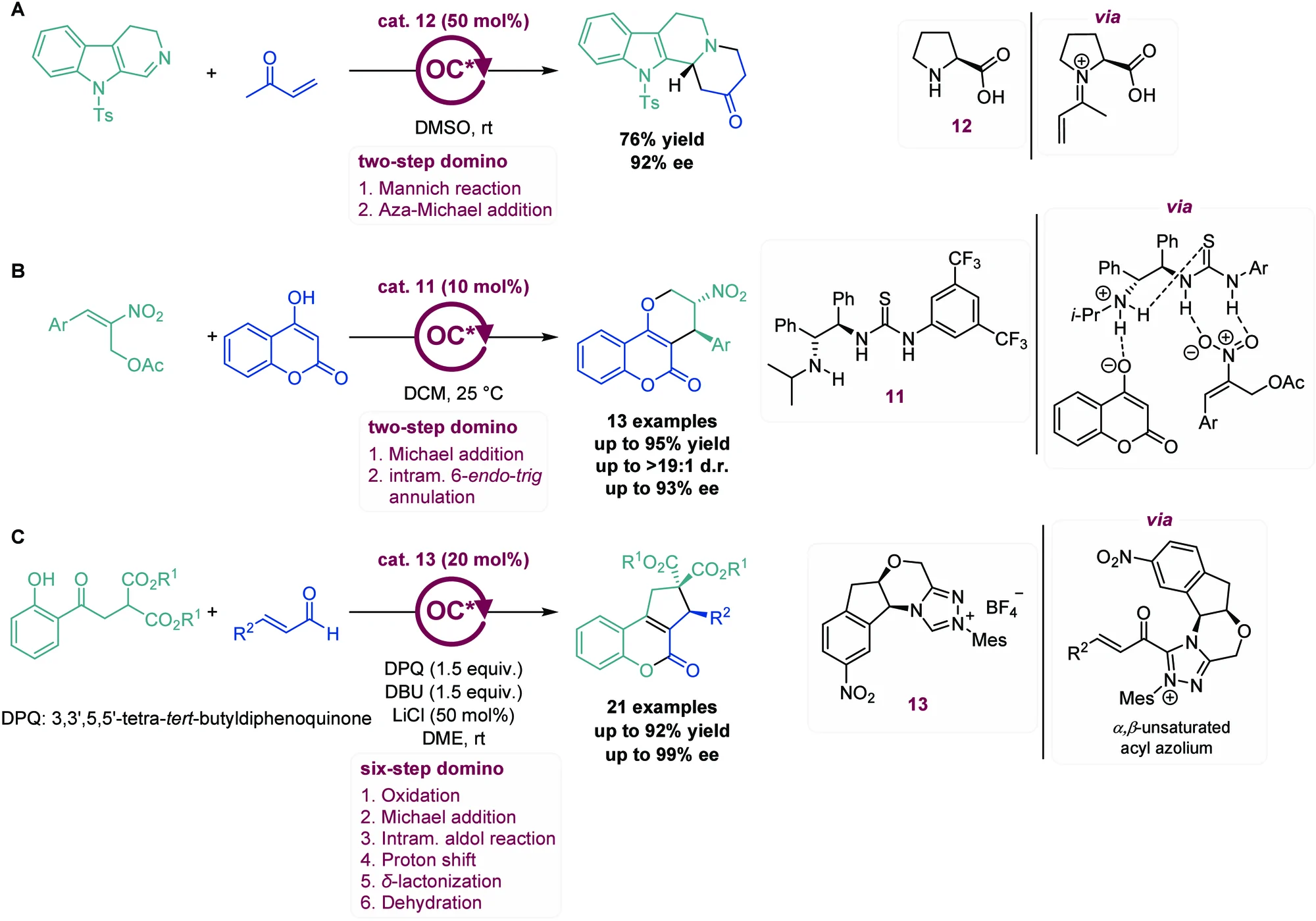
Enantioselective Synthesis of Multicyclic Heterocycles: A) Hexahydro-1*H*-indolo­[2,3-*a*]­quinolizin-2-one; B) Pyranocoumarins; C) Tricyclic Coumarin Derivatives

The enantioselective synthesis of pyranocoumarins using a bifunctional amine-thiourea organocatalyst was achieved by Li, Chen, and co-workers (Scheme 7B).^87^ Hydroxycoumarins and Morita–Baylis–Hillman acetate of nitroalkene react in a two-step domino reaction consisting of Michael addition and intramolecular 6-*endo-trig* annulation to produce biologically active pyranocoumarins in excellent yields and up to 93% ee.

Subsequently, Chen, Enders, and co-workers developed an *N*-heterocyclic carbene-catalyzed domino reaction toward tricyclic coumarin derivatives *via* α,β-unsaturated acyl azolium intermediates (Scheme 7C).^89^ The six-step domino reaction comprising oxidation/Michael addition/intramolecular aldol reaction proton shift/δ-lactonization/dehydration yields the coumarin derivatives in good-to-excellent yields and up to 99% ee. The increasing length and sophistication of domino sequences highlight both the opportunity and the intrinsic challenge of cascade chemistry: as complexity rises, so too does the possibility of competing pathways and diminished reproducibility.

### 3.4 Spirocyclic Heterocycles

An enantioselective synthesis of spirocyclic, polyfunctionalized cyclohexenes was reported by the Enders group (Scheme 8A).^90^ The organocatalyzed transformation features the possibility of oxidizing the resulting nucleophilic enamine with *o*-iodoxybenzoic acid (IBX) into the electrophilic iminium intermediate, enabling a four-step Michael addition/oxidation/Michael addition/aldol condensation domino reaction. The final products were obtained in good yields and up to >20:1 d.r. and 99% ee. The integration of oxidation steps using IBX within domino process expands synthetic flexibility, yet it also introduces considerations regarding reagent economy, sustainability, and waste generation.

**Scheme 8:**
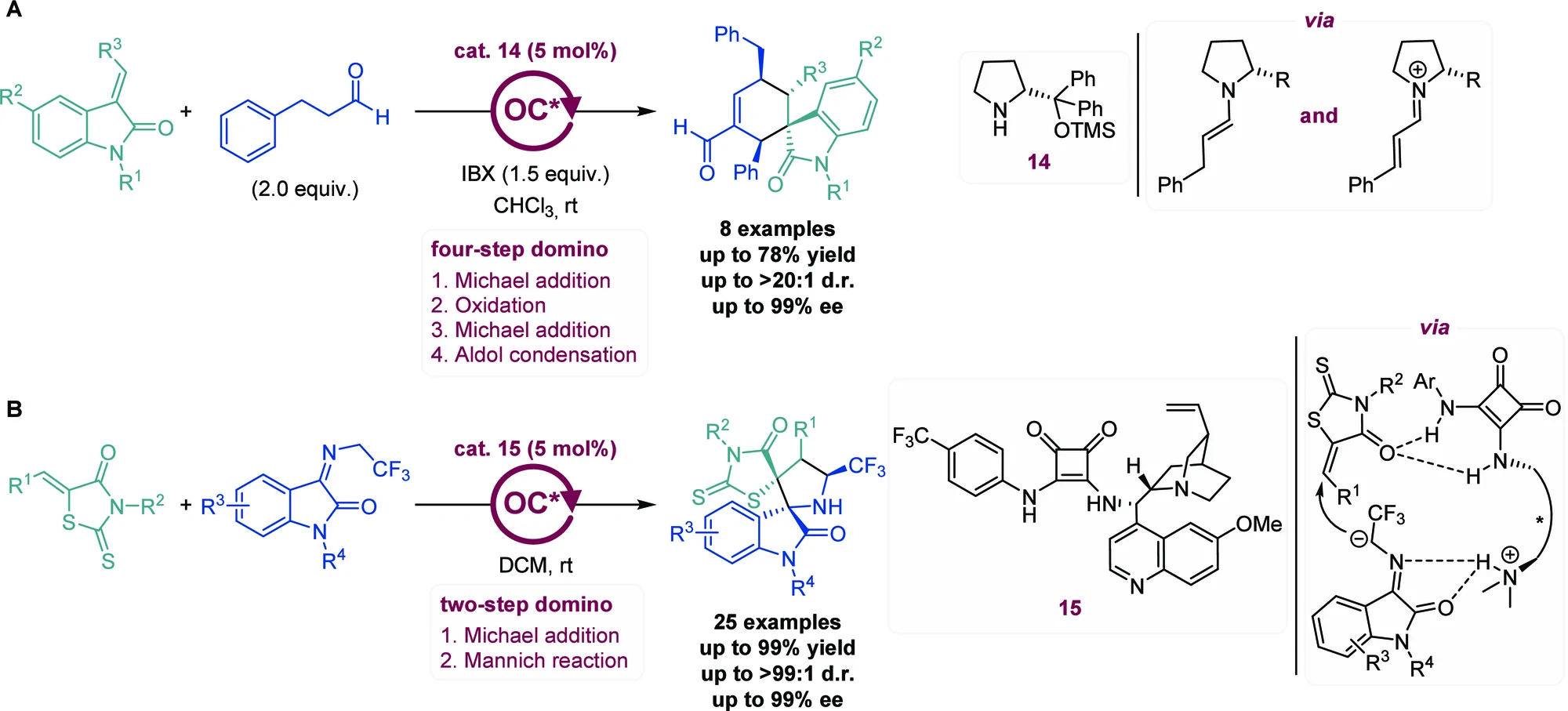
Enantioselective Synthesis of Spirocyclic Heterocycles: A) Spirocyclic Polyfunctionalized Cyclohexenes; B) Spirocyclic Functionalized Indolinones

The application of bifunctional organocatalyst in the enantioselective synthesis of spirocyclic derivatives was demonstrated by Du and co-workers (Scheme 8B).^91^ The two-step Michael/Mannich domino reaction between rhodanine derivatives and *N*-(2,2,2-trifluoroethyl)­isatin ketimines involves hydrogen-bonding and protonation interactions with the chiral bifunctional catalyst enabling the asymmetric synthesis of bispiro­[oxindole-pyrrolidine-rhodanine]­s in good to excellent yields and up to 99% ee. Spirocyclic frameworks remain highly attractive in drug discovery; continued efforts toward broadening functional-group tolerance will further enhance their practical value.

### 3.5 Bridged Heterocycles

An enantioselective reaction between *α,β*-unsaturated cyclic ketones, formaldehyde and aryl amines catalyzed by (*S*)-proline, was established by Sundén and the team to afford azabicyclic ketones. (Scheme 9A).^92^ The products were obtained in up to 90% yield and with up to 99% ee through a two-step domino reaction including a Mannich reaction and an intramolecular aza-Michael addition.

**Scheme 9:**
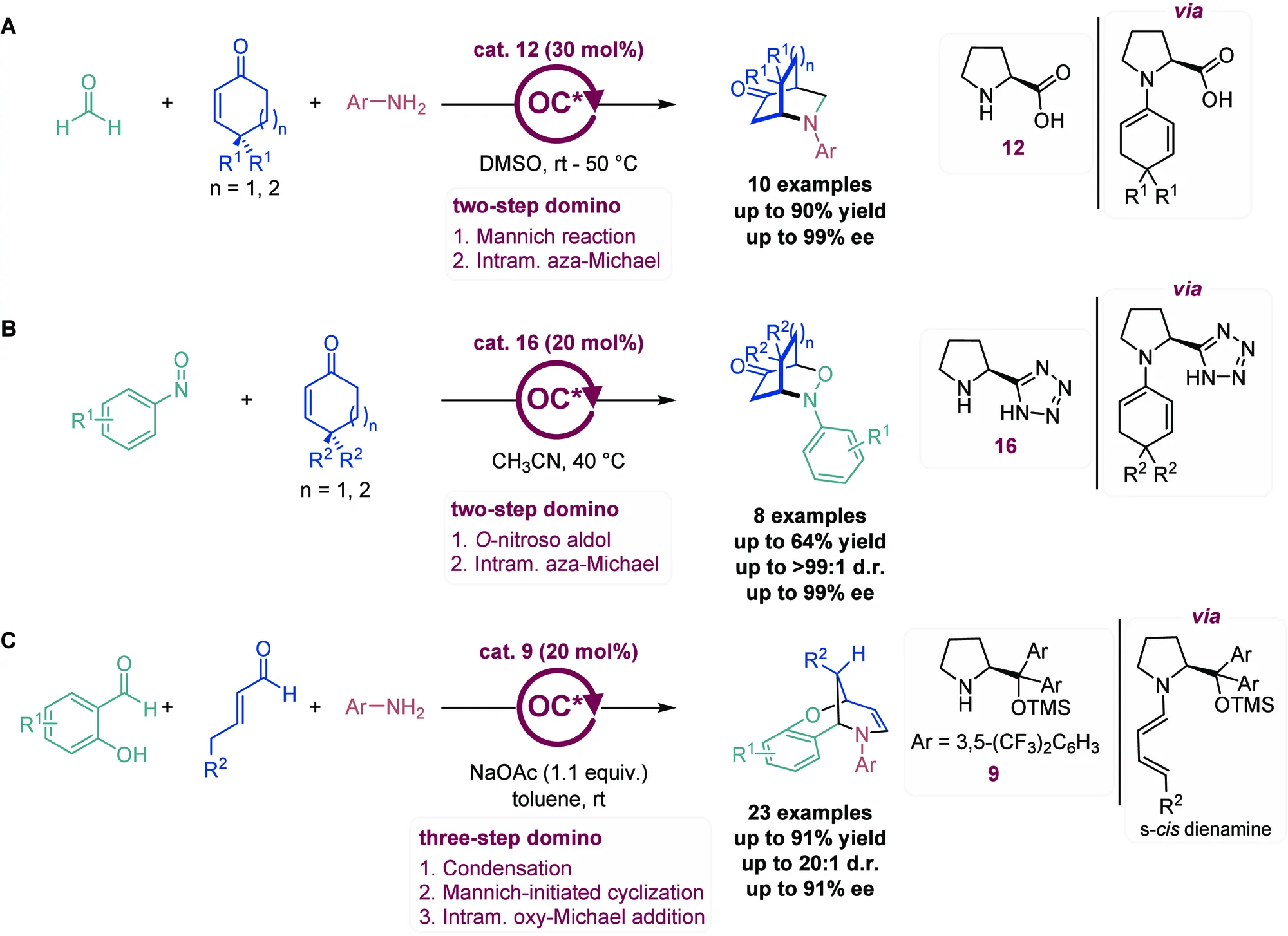
Enantioselective Synthesis of Bridged Heterocycles: A) Azabicyclic Ketones; B) Bicyclic Aminooxy Ketones; C) Bridged Benzoxazocines

Yamamoto and his working group reported a highly enantioselective synthesis toward a nitroso Diels–Alder adduct by applying a chiral pyrrolidine-tetrazole catalyst (Scheme 9B).^93^ In this process, nitrosobenzene and cyclic enone react *via* a two-step domino reaction comprised of *O*-nitroso aldol reaction and intramolecular aza-Michael addition, to produce eight derivatives with yields up to 64% and up to 99% ee. Although high enantioselectivity is achieved, improving yields and expanding substrate scope are necessary to enhance the broader applicability of such nitroso-based cascades. A novel asymmetric organocatalytic synthesis toward bridged benzoxazocines using *α,β*-unsaturated aldehydes, anilines, and electron-deficient salicylaldehydes was presented by Jørgensen, Albrecht and co-workers (Scheme 9C).^94^

The chiral pyrrolidine-catalyzed reaction proceeds *via* a three-step domino reaction consisting of condensation/Mannich-initiated cyclization/intramolecular *oxy*-Michael addition (Figure 6), furnishing the desired products in very good yields and high enantioselectivity of up to 91% ee.

**Figure 6:**
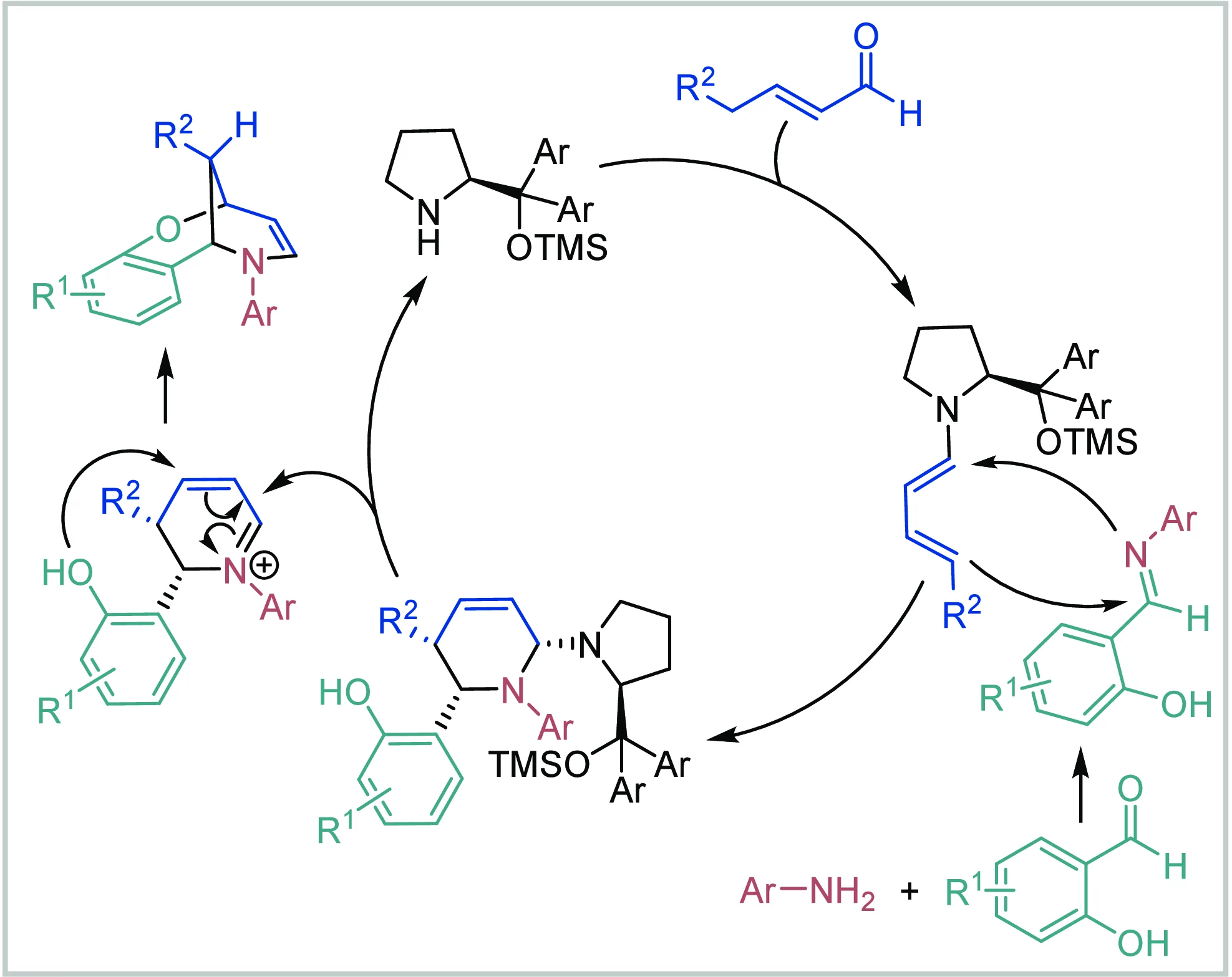
Proposed mechanism for the domino reaction presented in Scheme 9C.

Bridged systems represent some of the most demanding targets for asymmetric domino catalysis; future advances will likely require deeper mechanistic understanding, improved catalyst design, and enhanced predictability of cascade behavior.

All presented examples demonstrate the remarkable progress achieved in enantioselective organocatalyzed domino synthesis. In the broader quest for synthetic simplicity, such enantioselective methodologies demonstrate how molecular complexity can be constructed in a single operation, minimizing step count while maximizing structural elaboration. Yet the field now faces a new phase: moving from proof-of-concept demonstrations toward general, and scalable asymmetric domino processes. Achieving lower catalyst loadings, broader substrate generality, and industrial relevance will define the next generation of enantioselective domino strategies in the quest for synthetic simplicity.

## 4 Heteroaromatic Compounds

The domino synthesis of heteroaromatic compounds represents a particularly powerful strategy for the rapid assembly of conjugated and structurally rigid frameworks that are ubiquitous in bioactive compounds, natural products, and functional materials.^21,23,29,95^ In contrast to saturated heterocycles, the construction of aromatic systems often requires oxidative steps or elimination events, introducing additional redox processes that may complicate catalyst turnover and sustainability profiles. Thus, a central challenge in this area lies in balancing cascade efficiency with redox economy, operational simplicity, and avoidance of stoichiometric oxidants.

The organocatalyzed synthesis of bicyclic heteroaromatic compounds was demonstrated, for example, by the groups of Song and Chi reporting an NHC catalyzed reaction between diones and α,β–γ,δ-diunsaturated enals (Scheme 10A).^96^ Upon carbene addition, a key step for this transformation is a subsequent oxidation using 3,3′,5,5′-tetra-*tert*-butyldiphenoquinone (DPQ) for the generation of acyl azolium intermediate which enables the following 1,6-addition. The reaction encompasses an eight-step domino oxidation/1,6-addition/cyclizing intramolecular acylation/isomerization/aldol reaction/decarboxylation/oxidative aromatization process furnishing the 3-ylidenephthalides in good yields. The remarkable coordination of eight consecutive elementary steps within a single reaction flask highlights the conceptual elegance of domino design. However, the requirement for stoichiometric amounts of external oxidant DPQ (2.0 equiv) illustrates a limitation: redox-intensive cascades may compromise atom economy and generate additional waste. Future developments toward redox-neutral or catalytic oxidation protocols would significantly enhance the sustainability of such heteroaromatization strategies.

**Scheme 10:**
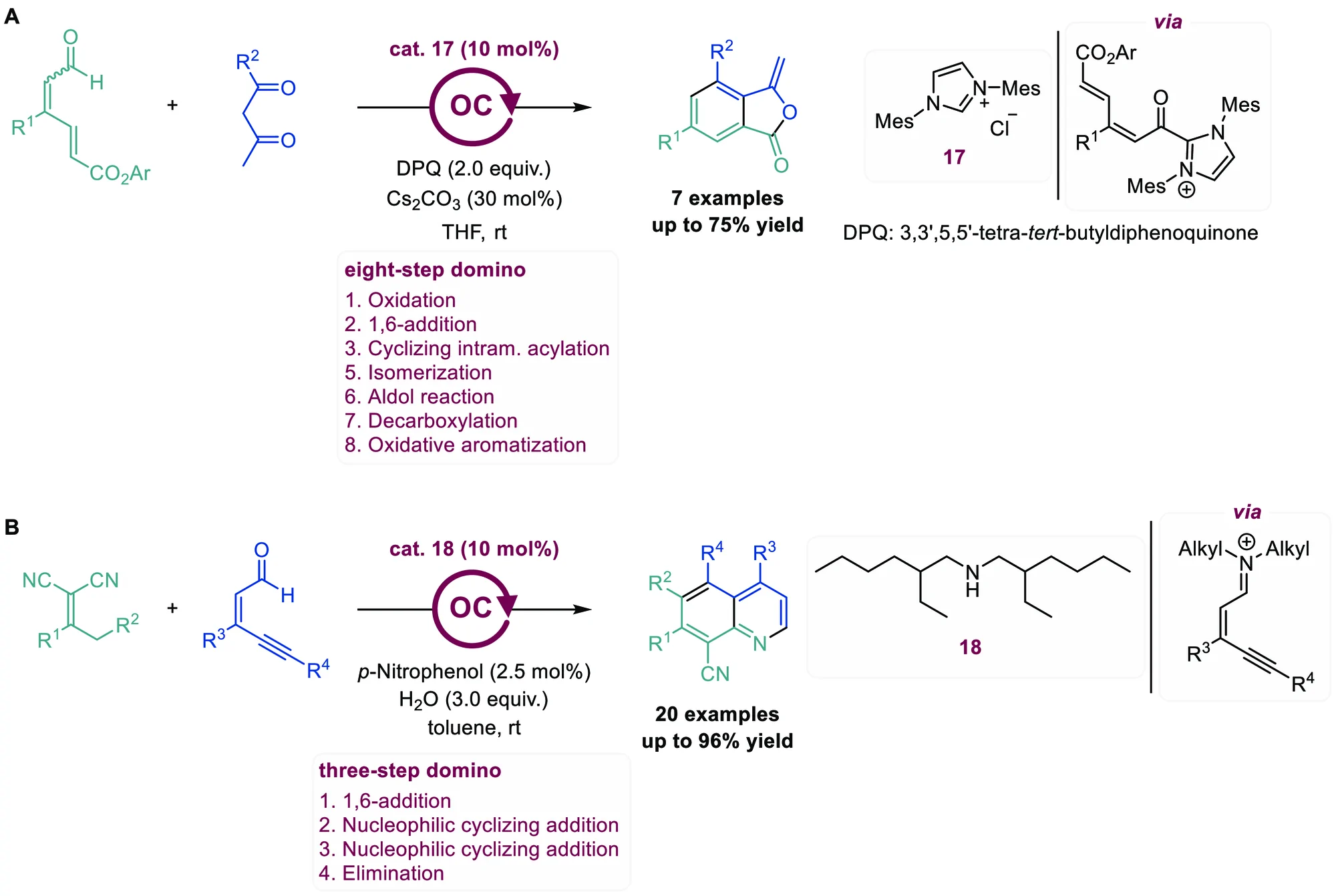
Synthesis of Multicyclic Heteroaromatic Compounds: A) 3-Ylidenephthalides; B) Substituted Quinolines

A secondary amine catalyzed synthetic route toward substituted quinolines was reported by the group of Hayashi (Scheme 10B).^97^ Herby, di­(2-ethyl)­hexylamine was employed as an organocatalyst for the four-step 1,6-addition/nucleophilic cyclizing addition/nucleophilic cyclizing addition/elimination domino reaction of dicyanoalkenes with enynals to afford the final products in good to excellent yields. This transformation demonstrates how simple amine catalysis can trigger complex annulation sequences leading to valuable quinoline scaffolds. Nevertheless, expanding the substrate scope beyond electronically activated partners and improving functional-group tolerance remain important objectives for broader synthetic applicability. Overall, the domino synthesis of heteroaromatic compounds vividly illustrates the dual nature of domino chemistry: unparalleled step economy on the one hand, but increasing mechanistic and redox complexity on the other.

As the field advances, key challenges will include minimizing stoichiometric oxidants, improving catalyst turnover, enhancing scalability, and ensuring predictable domino reaction behavior. Ultimately, the development of redox-economical and operationally robust domino aromatization processes will be essential for translating these elegant laboratory demonstrations into broadly applicable industrial synthetic tools.

## 5 Total Synthesis of Bioactive Heterocycles *via* Domino Reactions

In the context of total synthesis, domino reactions play a particularly strategic role, as a single domino transformation can determine the overall efficiency and practical feasibility of an entire synthetic route.^28^ Several contributions by Tsogoeva group have already demonstrated the integration of organocatalyzed domino processes with one-pot strategies for the synthesis of bioactive heteroaromatic scaffolds, including quinazolines^95,98–100^ and phthalazines^101^ with antiviral and anticancer properties. Furthermore, the combination of asymmetric organocatalyzed domino reactions with one-pot protocols has been effectively exploited for the enantioselective multistep synthesis of diverse heterocycles by several research groups, including those of Jørgensen,^102–104^ Hayashi,^105,106^ List,^107^ and Lattanzi.^108^ Such approaches enable the rapid construction of complex molecular frameworks while minimizing purification steps and improving step economy. Organocatalyzed domino reactions have therefore become valuable tools for the synthesis of key heterocyclic motifs within complex natural products. Their utility has been demonstrated in several total syntheses, including those of the potent cytotoxic alkaloid camptothecin (Scheme 11A) and natural product 4-dehydroxydiversonol (Scheme 11B).

**Scheme 11:**
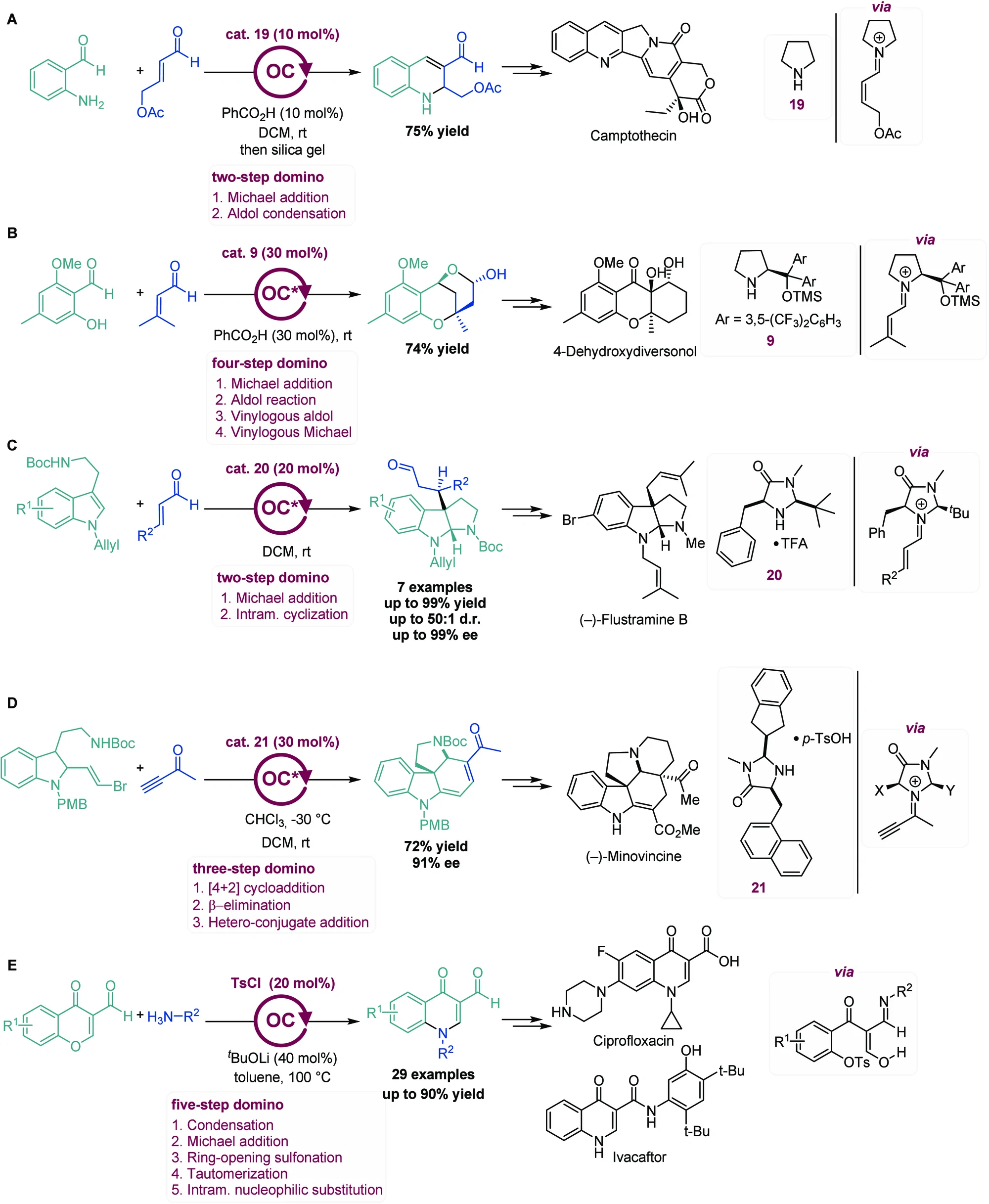
Total Synthesis of Bioactive Heterocyclic Compounds *via* Organocatalysis. Entry to A) Camptothecin; B) 4-Dehydroxydiversonol; C) (−)-Flustramine B; D) (−)-Minovicine; E) Ciprofloxacin and Ivacaftor

An important dihydroquinoline precursor for the synthesis of the anticancer agent camptothecin was reported by the group of Yao starting from simple substrates *via* an organocatalyzed domino reaction (Scheme 11A).^109^ The pyrrolidine-catalyzed two-step Michael addition/aldol condensation domino reaction allowed for the formation of the dihydroquinoline derivative in 75% yield. This intermediate can be further modified toward naturally occurring camptothecin in 13 steps. While the domino step efficiently establishes the core dihydroquinoline scaffold, the overall sequence still requires multiple transformations, illustrating that even highly effective domino reactions must be integrated thoughtfully into longer synthetic plans to maximize overall step economy.

Bräse and co-workers established an asymmetric organocatalyzed reaction to a precursor for the total synthesis of the natural product 4-dehydroxydiversonol (Scheme 11B).^110^

The transformation of salicylaldehydes and senecialdehydes using Jørgensen catalyst proceeds through a four-step Michael addition/aldol reaction/vinylogous aldol reaction/vinylogous Michael domino process to yield the precursor in 74%. After 5 additional steps, 4-dehydroxydiversonol can be obtained. This example highlights the ability of asymmetric organocatalysis to construct functionalized frameworks rapidly. Nevertheless, further reducing catalyst loading and improving turnover numbers will be crucial for industrial implementation.

Starting from tryptamines and *α,β*-unsaturated aldehydes, the group of MacMillan developed an enantioselective organocatalytic method for the synthesis of pyrroloindolines using a chiral imidazolidinone catalyst. These scaffolds represent valuable structural motifs for the total synthesis of the natural product (−)-flustramine B (Scheme 11C).^111^ Seven derivatives were generated with excellent yields and enantioselectivities of up to 99% ee *via* a two-step Michael addition/intramolecular cyclization domino reaction. Later on, MacMillan and co-workers also developed an enantioselective approach to an important precursor of (−)-minovicine (Scheme 11D).^112^ With the use of a chiral amine catalyst, 3-butyn-2-one and a vinyl indole derivative form the product *via* a three-step [4 + 2] cycloaddition/beta-elimination/heteroconjugate addition domino reaction with a yield of 72% yield and 91% ee. The success of these strategies shows how asymmetric organocatalyzed domino processes enable rapid access to complex bioactive frameworks; however, extending such methodologies to more structurally diverse natural product families remains an open frontier.

Recently, a TsCl-mediated reaction toward a synthetically valuable 4-quinolone derivative was reported by the group of Xu (Scheme 11E).^113^ Chromone-3-carboxaldehydes and amines undergo a five-step condensation/Michael addition/ring-opening sulfonation/tautomerization/intramolecular nucleophilic substitution domino reaction to afford precursors for quinolone-based antibiotics such as ciprofloxacin, perfloxacin and norfloxacin, as well as for the cystic fibrosis drug ivacaftor, in very good yields of up to 90%.

Although domino reactions efficiently deliver pharmaceutically relevant intermediates, the use of activating reagents such as TsCl raises considerations regarding reagent economy and waste generation. Future developments aimed at milder, catalytic activation modes would further strengthen the sustainability profile of such transformations. All these examples demonstrate that organocatalyzed domino reactions have moved beyond methodological curiosity and now occupy a strategic position in total synthesis. The next phase of development will require not only high selectivity and elegance, but also robustness, scalability, reduced catalyst loading, and improved sustainability metrics. Ultimately, the true measure of success will lie in whether domino organocatalysis can consistently shorten synthetic routes, lower costs, and enable more sustainable access to bioactive heterocyclic compounds on practical scales. Translating elegant organocatalyzed domino processes from model systems to the demanding applications in industrial synthesis, especially for complex bioactive heterocycles, and drug-like scaffolds, remains a significant challenge, particularly with respect to scalability, robustness, and functional-group compatibility.

Ultimately, the true measure of success will lie in whether domino organocatalysis can consistently shorten synthetic routes, lower costs, and enable more sustainable access to bioactive heterocyclic compounds on practical scales.

## 6 Light-Induced Domino Reactions toward Heterocyclic Compounds

A synthetically attractive approach for constructing heterocycles is the combination of organocatalysis and photoredox catalysis. The merger of light activation with organocatalytic activation modes has opened access to radical reactivity patterns that are difficult to achieve under purely thermal conditions,^114^ thereby significantly expanding the synthetic toolbox for heterocycle construction. At the same time, the coordination of dual catalytic cycles introduces challenges in catalyst compatibility, reaction control, and scalability.

An interesting example for combining organocatalysis with photocatalysis was presented by Melchiorre and co-workers (Scheme 12A).^115^ A dual chiral Lewis base catalysis and photocatalysis enabled the first enantioselective radical conjugate addition to α,β-unsaturated anhydrides and esters leading ultimately to enantioenriched pyrrolidinones. The method demonstrates an enantioselective radical pathway encompassing a six-step nucleophilic addition/decarboxylation/radical conjugate addition/reduction/radical-polar crossover/intramolecular acylation domino process. The desired products were obtained in good yields and excellent enantiomeric ratios (up to 94:6 e.r.). This work highlights the conceptual power of combining photochemical single-electron transfer with asymmetric organocatalysis to control stereochemistry in radical cascades.

**Scheme 12:**
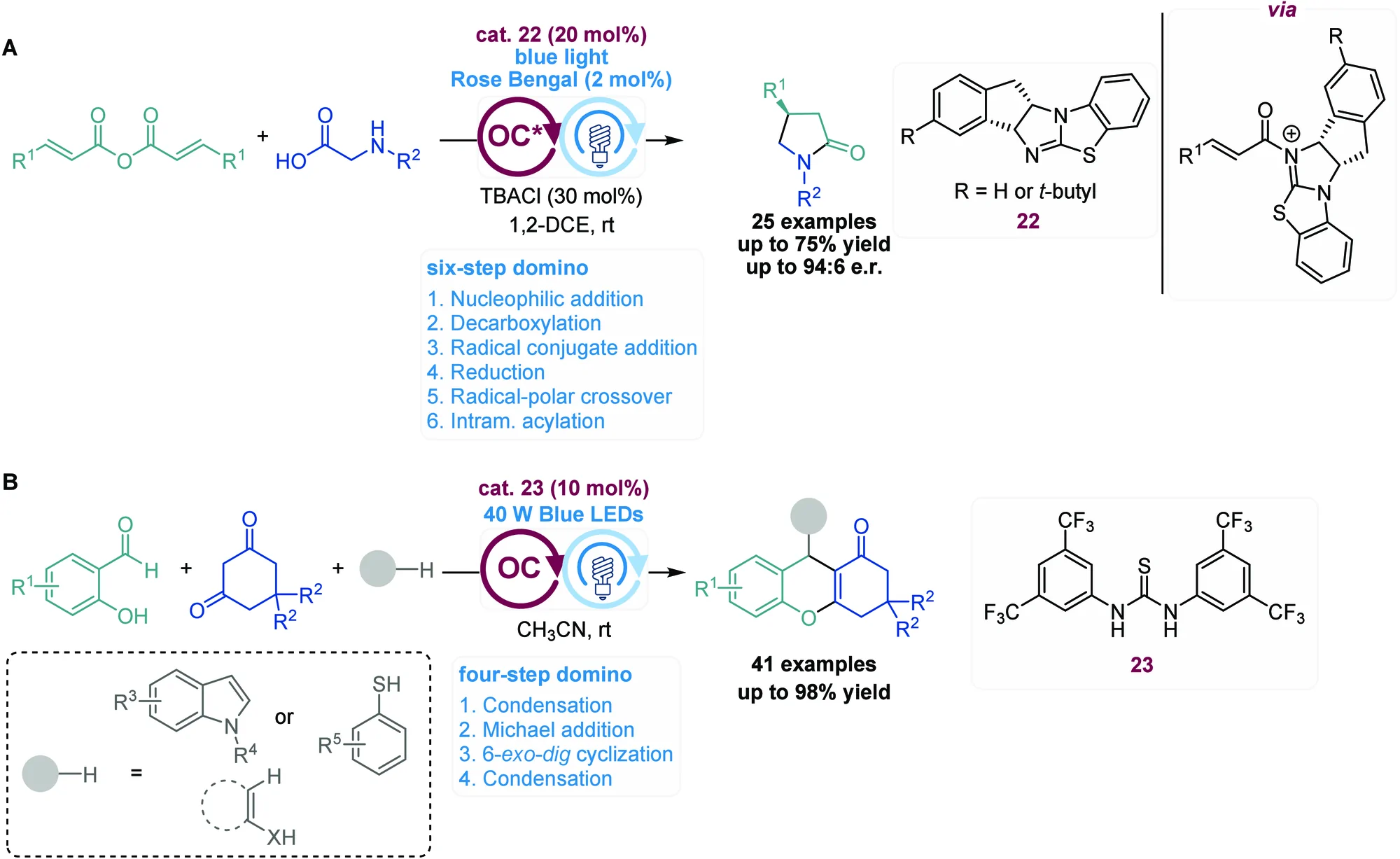
Light-Induced Domino Reactions Toward Heterocyclic Compounds: A) Pyrrolidinones; B) Xanthenes

A quite different approach was presented by Chowhan, Borah and co-workers, reporting a dual organocatalytic and photochemical protocol for the C–H and S–H functionalization of bioactive compounds (Scheme 12B).^116^ The organocatalyzed three-component reaction between cyclic-1,3-diketones, 2-hydroxybenzaldehydes and the respective C–H or S–H derivative proceeds *via* the photoexcitation of substrate-catalyst’s hydrogen-bonding complex as key step. The four-step condensation/Michael addition/6-*exo-dig* cyclization/condensation domino reaction furnishes the functionalized xanthene scaffolds in good to excellent yields. This strategy demonstrates that light-induced activation can bypass the need for external photocatalysts by exploiting photoactive hydrogen-bonded assemblies, thereby simplifying reaction design. However, broader substrate scope, and mechanistic understanding of photoexcited complexes remain important areas for future investigation.

Overall, light-induced domino reactions represent a rapidly evolving frontier in heterocyclic synthesis. Their capacity to merge radical and polar reactivity within a single catalytic manifold offers unprecedented synthetic opportunities. Future progress will likely focus on improving reaction efficiency, expanding enantioselective variants, developing continuous-flow photoreactors, and enhancing the sustainability profile of these transformations to enable practical and large-scale industrial implementation.

## 7 Autocatalyzed Domino Reactions toward Heterocyclic Compounds

Autocatalysis refers to a process in which a reaction product promotes its own formation, creating a lower-energy pathway that leads to characteristic self-accelerating kinetics after an initial induction period.^117,118^ This behavior is particularly significant in asymmetric synthesis exemplified by the Soai reaction, which can amplify enantiomeric excess and is proposed to proceed *via* quadratic autocatalysis by homochiral Zn-complexes as the active catalysts.^119–122^ Consequently, autocatalysis has attracted growing attention in synthetic organic chemistry, as mechanistic insights into such processes can facilitate the optimization of catalytic transformations while also reducing waste, since no external catalyst is required.

Photo- and organo-autocatalyzed domino reactions represent recent advances—an extension of classical autocatalysis—and constitute an elegant evolution of catalyzed domino chemistry. In these systems, the substrate, an intermediate, or an *in situ-*generated species actively contributes to driving the transformation forward.^123,124^ By eliminating the need for external photocatalysts or organocatalysts, the self-catalysis strategies—photoautocatalysis^124^ and organo-autocatalysis^125^—align closely with the principles of step economy, catalyst economy, and sustainability. However, achieving efficient and controlled self-catalysis requires precise substrate design and a deep mechanistic understanding of reactive intermediates. Against this backdrop, photo*-* and organo-autocatalytic systems have emerged as particularly promising approaches, as demonstrated by recent studies.

### 7.1 Photoautocatalysis

Recently, the Boddaert group reported a photoautocatalyzed domino reaction involving photoexcitation of pyrenacyl sulfides, followed by fragmentation to thiocarbonyls and 1-acetylpyrene (Scheme 13A).^126^ The latter serves also as a photocatalyst and, upon its excitation, undergoes an energy transfer step with the respective thiocarbonyls to form the excited species required for the final cyclization toward substituted thietane derivatives. The reaction encompasses two-step Norrish type II fragmentation/thia-Paternò-Büchi domino process and affords the final products in good to excellent yields and up to >20:1 diastereomeric ratio. This example demonstrates how *in situ*-generated photoactive species can sustain a catalytic cycle, reducing reliance on external photocatalysts.

**Scheme 13:**
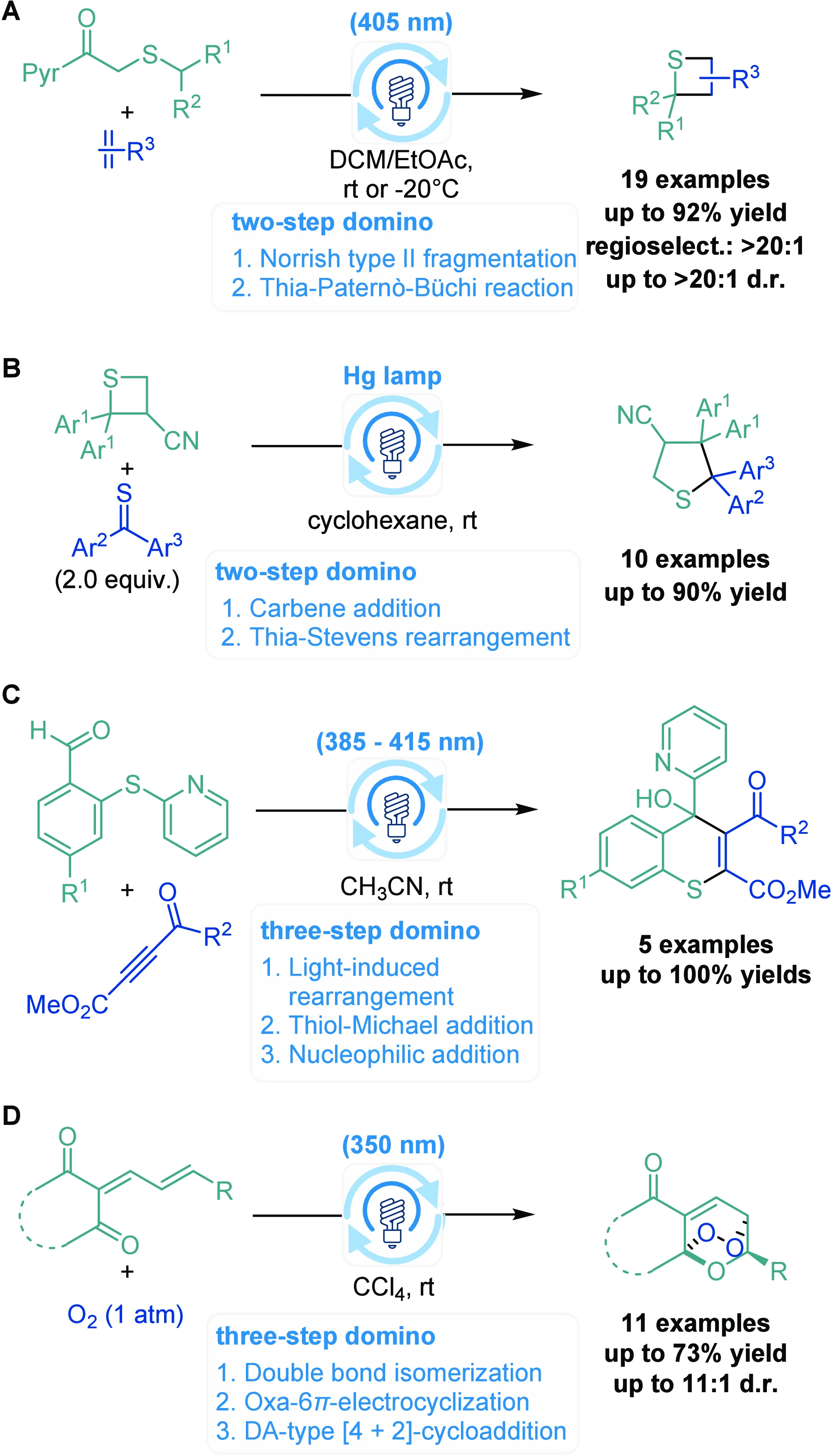
Self-Catalyzed Photochemical Domino Reactions Toward A) Thietan Derivatives; B) Tetrahydrothiophenes; C) Thiochromene Derivatives; D) Trioxane Compounds

Boddaert, Aitken and co-workers developed a photoautocatalyzed reaction toward tetrahydrothiophenes starting from thietane and photoexcited thiocarbonyl substrates (Scheme 13B).^127^ The ring enlargement is attributed to a two-step domino reaction including carbene addition and thia-Stevens rearrangement (Figure 7A), to afford the products in up to 90% yield. Such two-step domino process illustrates the potential of photochemical activation to access functionalized cyclic organosulfur compounds; nevertheless, controlling selectivity in highly reactive carbene pathways remains a key challenge for broader application.

**Figure 7:**
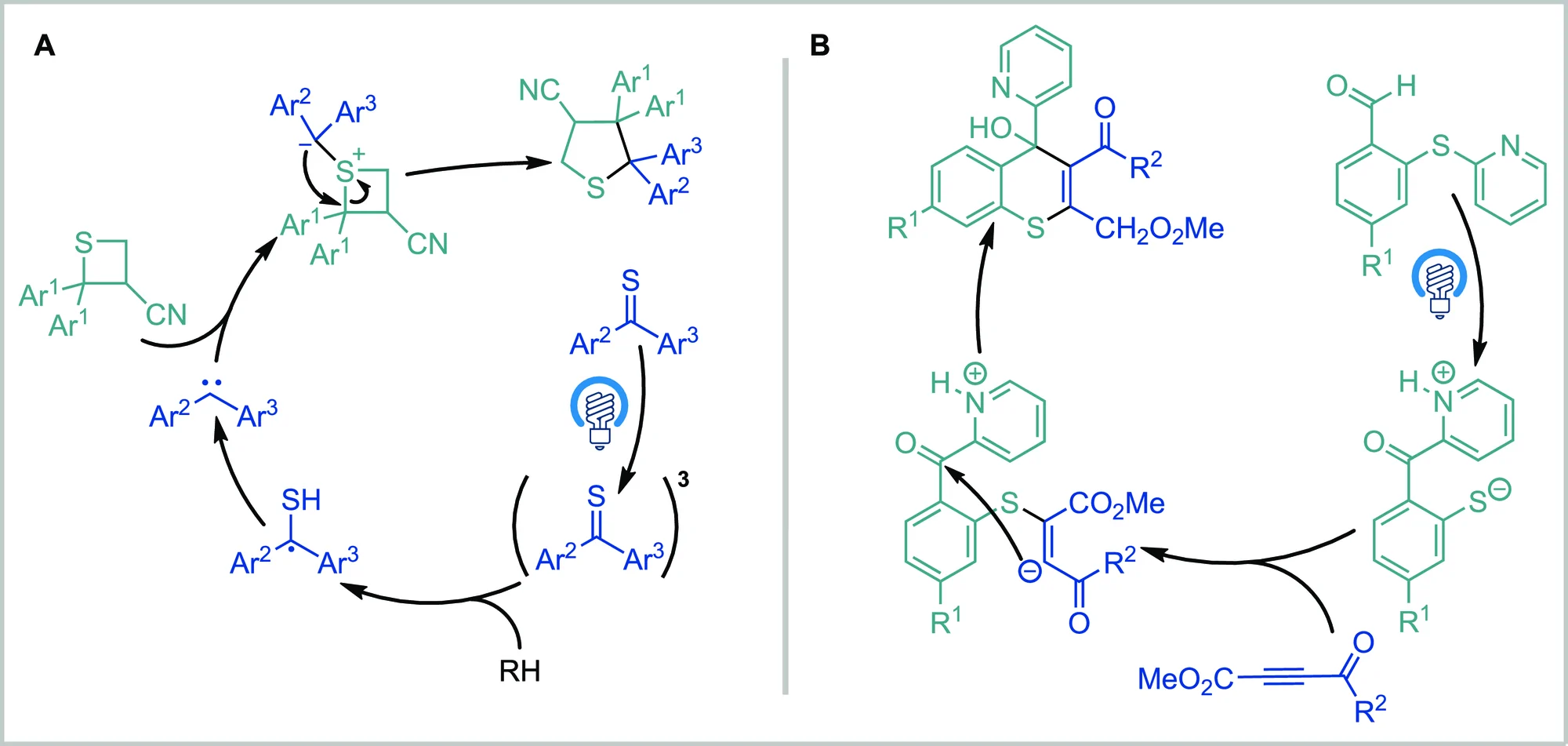
Plausible reaction mechanisms for the domino transformations presented in Scheme 13B (A) and Scheme 13C (B).

Further approaches also exploit the intrinsic photoactivity of substrates to mediate photochemical transformations, thereby eliminating the need for an external photocatalyst. An example of such photoautocatalyzed domino reactions was presented by Feist, Barner-Kowollik and co-workers (Scheme 13C).^128^ The light-excitation of *o*-thiopyrinidylbenzaldehydes initiates a three-step light-induced rearrangement/thiol-Michael addition/nucleophilic addition domino process (Figure 7B) furnishing the final products in up to quantitative yields.

In a similar manner, the photochemical excitation of allylidene-1,3-cycloalkanediones under aerobic conditions was investigated by Mischne and co-workers (Scheme 13D).^129^

The work exploits the self-sensitization properties of starting material to generate singlet oxygen species involved in the three-step double bond isomerization/oxa-6π-electrocyclization/Diels–Alder-type [4 + 2]-cycloaddition domino synthesis of 1,2,4-trioxanes. The products were obtained in good yields and diastereomeric ratios. Harnessing molecular oxygen as a traceless oxidant further strengthens the sustainability profile of such processes, though improved control over oxygen concentration and photochemical reactor design will be essential for safe scale-up.

Overall, the avoidance of external metal-based or organic photocatalysts simplifies purification and reduces potential metal contamination, which is particularly attractive for pharmaceutical industry. However, substrate-specific photoactivity can limit general applicability, making rational chromophore design an important future direction.

Future advances will likely focus on expanding substrate scope, and harnessing self-catalysis to provide cost-efficient, sustainable, and practical access to bioactive heterocyclic compounds in both academic and industrial settings.

### 7.2 Organo-autocatalysis

Early studies on non-enantioselective organo-autocatalytic reactions demonstrated that organic products can act as their own catalysts.^130,131^ The first example of enantioselective organo-autocatalysis was demonstrated in a Mannich reaction, in which the chiral product catalyzed its own formation.^132^ Subsequent studies confirmed the role of the Mannich product as an organo-autocatalyst, with yields and stereoselectivities strongly dependent on the solvent.^133^ Further investigations of the Mannich reaction system using the authentic products and also product mimics as organo-autocatalysts, revealed changes in stereochemical purity during the reaction and highlighting epimerization effects that influence the final diastereomeric ratio.^134^

Over recent years, organo-autocatalysis has emerged as a sustainable, highly atom-economic, and cost-efficient approach for promoting organic transformations.^125^ Building on these bases, the scope of one-step organo-autocatalysis has recently been expanded to encompass multistep domino reaction mechanisms. Tsogoeva and co-workers disclosed an organo-autocatalyzed transamination metathesis of two C­(sp^2^)–N bonds in a cyclic substrate relying on the *in situ* formation of a pyrrolidinium salt, acting as an organo-autocatalyst (Scheme 14A).^135^ This allows for the challenging transformation to take place under exceptionally mild reaction conditions at room temperature without external catalysts and/or additives. The role of the *in situ*-formed pyrrolidinium salt in the catalytic cycle lies in forming hydrogen-bond interactions with the substrate, enabling a six-step aza-Michael addition/ring-opening reaction/tautomerization/intramolecular aza-Michael reaction/intramolecular S_N_2 reaction/elimination domino process (Figure 8). The products were obtained in good to excellent yields of up to 95%.

**Scheme 14:**
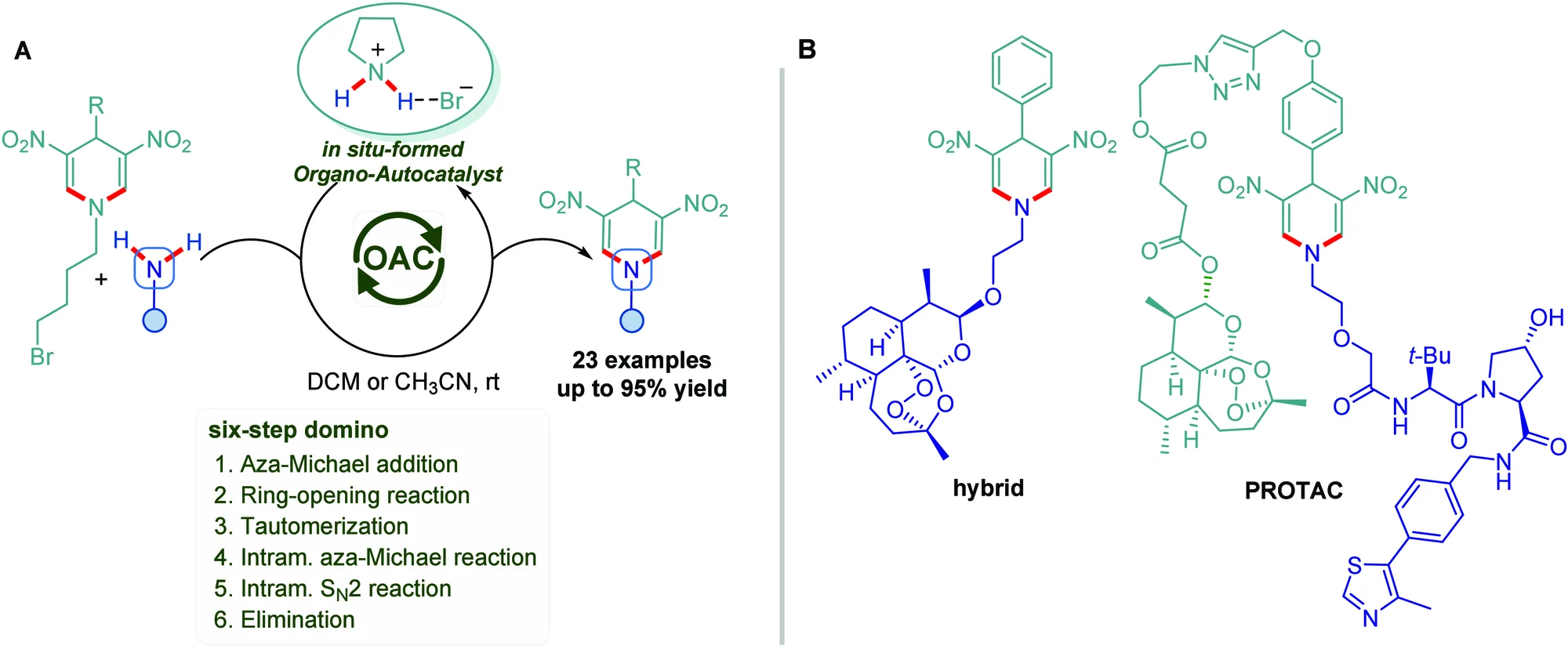
A) Organo-Autocatalyzed Domino Reaction Toward Dihydropyridines; B) Selected Examples of Bioactive Products

**Figure 8:**
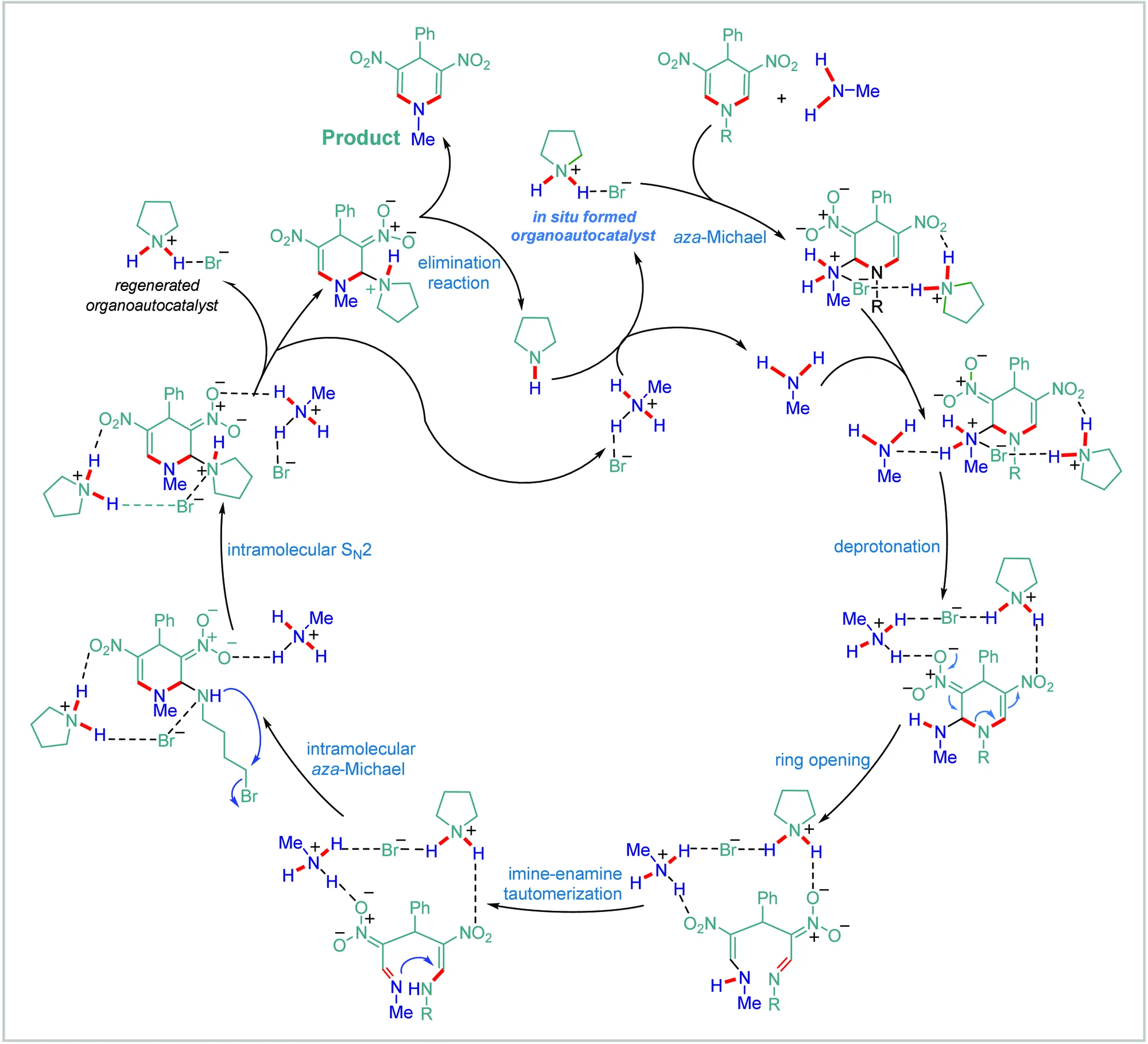
DFT-calculated catalytic cycle for the organoautocatalyzed transamination metathesis reaction.

This organo-autocatalyzed reaction gives rapid, environmentally friendly, and atom-economical access to a broad range of N-substituted 1,4-dihydropyridines (1,4-DHPs), thus offering facile and high-yielding entry to privileged structures in pharmaceuticals and bioactive compounds, e.g., antiviral artemisinin-based hybrids and PROTACs (Proteolysis Targeting Chimeras) (Scheme 14B).^135^ More broadly, this example underscores the synthetic potential of self-sustaining and autocatalytic processes. Organo-autocatalysis—a specific type of autocatalysis in which the catalyst is an organic molecule produced by the reaction itself—is emerging as a versatile platform for designing new highly sustainable reaction systems. Such organo-autocatalytic strategies highlight a particularly appealing concept: the *in situ* generation of a catalytic species from the applied reagents, thereby achieving maximal atom and catalyst economy. Moving forward, further discoveries and developments in this field are expected, and expanding organoautocatalytic designs to a broader range of heterocyclic compounds might enhance their synthetic impact.

Organo-autocatalysis—a specific type of autocatalysis in which the catalyst is an organic molecule produced by the reaction itself—is emerging as a versatile platform for designing new highly sustainable reaction systems.

## 8 Outlook

Advances in organo- and photocatalytic domino reactions over the past two decades have clearly demonstrated their power and ability to rapidly construct complex heterocyclic scaffolds with excellent chemo-, regio-, and stereocontrol. However, several challenges still need to be addressed before these methods can reach their full potential in industrial settings. While remarkable efficiencies have been achieved at laboratory scale, broader substrate scope, reduced catalyst loading, improved turnover numbers, and enhanced robustness under operationally simple conditions remain highly desirable goals.

One critical direction concerns catalyst economy. Many highly selective organocatalytic domino reactions still rely on relatively high catalyst loadings or structurally elaborate chiral scaffolds. Future efforts should focus on identifying more active and recyclable catalytic systems, or minimalistic catalysts capable of maintaining selectivity while improving atom and cost efficiency.

A second important aspect is energy efficiency and reaction design. The fast growth of light-driven domino reactions shows the potential of photochemistry to access new radical–polar reaction pathways. However, issues such as efficient use of light (photon economy), scaling up in continuous-flow photoreactors, and reactor standardization are still less developed than for thermal reactions. Combining flow chemistry with better light sources and energy-efficient setups will likely be key for the next stage of practical applications.

Third, reaction robustness and sustainability should be key priorities in developing new methods. Some important questions to consider include:

1) Can changes in solvent, concentration, or reaction setup lower the material input?
2) Are external oxidants or activating reagents really necessary, or can they be replaced by traceless or catalytic alternatives (e.g., oxygen, electricity, or light)?
3) How tolerant is the reaction to air, moisture, and nonpure (technical-grade) reagents?
4) Can we reduce the amount of catalyst without losing selectivity?
5) Can we measure and compare the environmental impact of the domino transformation?

Forth, expanding the scope of domino synthesis toward increasingly complex, biologically relevant, and industrially meaningful targets will be decisive. The true test of these methodologies lies not only in reaction novelty, but in their capacity to shorten synthetic routes, reduce purification steps, and improve overall process sustainability.

Finally, an important question is whether a given domino transformation can be performed using photo*-* or organo-autocatalysis. Moving from classical organo- and photo-catalysis toward organoauto- and photoauto-catalytic systems, in which catalytic species are generated *in situ* from substrates or intermediates, represents an especially promising emerging research direction for minimizing external inputs and reducing costs and waste. New discoveries and developments are anticipated in the near future, and we envision that the field of organocatalysis will be complemented by organo-/photoautocatalysis, thereby also complementing the already well-established biocatalysis, transition metal catalysis, and photo/electrocatalysis.

Moving from classical organo- and photo**-**catalysis toward organoauto- and photoauto-catalytic systems, in which catalytic species are generated *in situ* from substrates or intermediates, represents an especially promising emerging research direction for minimizing external inputs and reducing costs and waste.

Organo-/photoautocatalyzed domino reactions embody one of the most sustainable directions in modern synthetic chemistry. By minimizing external inputs and leveraging intrinsic substrate reactivity, this strategy challenges traditional catalyst paradigms. Future research will need to address mechanistic predictability and broader substrate compatibility, and ultimately determine whether self-sustaining domino processes can transition from conceptual elegance to routine synthetic methodology in academia and industry.

## 9 Conclusions

In this Outlook, we have highlighted the evolution of domino synthesis of complex heterocycles, tracing its development from classical organo-/photo-catalysis to emerging concepts of photo-autocatalysis and organo*-*autocatalysis. These strategies exemplify how multiple bond-forming events can be controlled in a single operation, offering exceptional step economy and rapid access to sophisticated heterocyclic frameworks.

We have showcased advances in non-enantioselective and enantioselective organocatalytic multicomponent domino reactions, light-induced processes, and photo-/organo-autocatalytic systems that minimize or eliminate external catalytic inputs.

These approaches show that domino reactions are no longer just academic curiosities but are becoming valuable tools in total synthesis, medicinal chemistry, and the preparation of functional materials.

At the same time, important limitations remain. Factors such as catalyst cost and loading, reaction scalability, energy use, solvent choice, and downstream processing all affect the overall sustainability of a domino transformation. In many areas of synthetic chemistry, reaction novelty has historically been prioritized over environmental impact. However, the conceptual groundwork now exists to incorporate sustainability considerations directly into domino reaction design.

We envision that the next generation of domino methodologies will combine multiple green design elements at once: low-loading or *in situ*-generated organo-/photo-catalysts, benign solvents, energy-efficient activation methods, and robust, scalable conditions. In this way, domino synthesis can move from elegant laboratory reactions toward broadly applicable, industrially relevant technologies. Ultimately, by embracing the principles of step economy, catalyst economy, and reaction simplicity, domino strategies may in the future play a central role in enabling more sustainable, waste-reducing access to complex heterocyclic architectures.

## Supplementary Material



## Notes

Transparent Peer Review report available (PDF)
