# Supplementary material for: Domino Strategies in Heterocycle Synthesis: Advancing from Organocatalysis to Photo-/Organo-Autocatalysis
Source: ACS Cent Sci. 2026 Jul 13;12(8):1068–88. doi: 10.1021/acscentsci.6c00561 (PMC13587925; doi:10.1021/acscentsci.6c00561)
Supplement: Supplementary file 1 [file oc6c00561_si_001.pdf]

oc-2026-005613.R1

Name: Peer Review Information for "Domino Strategies in Heterocycle Synthesis: Advancing from Organocatalysis to Photo-/Organo-Autocatalysis"

First Round of Reviewer Comments

Reviewer: 1

Comments to the Author

The outlook by Tsogoeva et all. describes domino strategies to access heterocyclic compounds with a particular focus on organocatalysis and photo-/organo-autocatalysis. The outlook is structured in 7 sections plus an outlook and a conclusion. The sections are well organized and selected examples are described within the evolution of domino synthesis.

The outlook deserves to be published after careful revisions of the following points.

Major revisions:

- In an attempt to be concise, the authors only depicted the general schemes of the reactions, summarising the sequence of the domino reactions. However, since domino reactions may have a complex mechanism, I would encourage the authors to reconsider the schemes and provide more information about the reaction mechanisms. For example, I would suggest:

Scheme 2A, add at least the structure of the acyl azolium intermediate

Scheme 3B, add the structure of the intermediate characterized by polarity inversion

Scheme 6B, add the structure of the reactive trienamine species

Scheme 6D, add the iminium-allenamine cascade, since it defined unprecedented

Scheme 7C, add the structure of the acyl azolium intermediate

Scheme 12, please add the structure of the excited state required for final cycloaddition

Minor revisions:

-Page 2 line 53; please correct cinchona with Cinchona

-Page 5 line 45, right column, please correct 4-m ercapto-2-butenoate with 4-mercapto-2-butenoate

-Page 9 line 40, left column, please correct Nitrosobenzene with nitrosobenzene

Page 16, right column, the first paragraph is in the wrong column.

-The text should be carefully revised to uniform formatting of word that should be written in italics like (N-; trig, tert-), like pages 5, 10 and 16 plus schemes 7, 10 and 11E

-Please specify the acronym DAR (scheme 13 and page 15), MBH page 7

-Scheme 4, please add the structure of carbobicycle

-Scheme 5, the font size of the catalysts is bigger than the other text; please correct

-Scheme 9A, please correct the structure of the product

-Please remove Figure 2, it does not add anything to the outlook

Reviewer: 2

Comments to the Author

This Outlook highlights progress over time in enantioselective domino reactions, specifically focusing on heterocyclic and heteroaromatic compounds construction, their total synthesis, and self-catalyzed processes. By examining these transformations under organo- and photo-promoted conditions, this Outlook addresses a topic of significant relevance and interest to organic, medicinal, materials and industrial chemists. The manuscript, after a general introduction of the topic, has been sectioned starting from “Non enantioselective synthesis of heterocycles” then sectioned according to the mon-, multiand bridged- heterocycles and so on including stereoselective variants, illustrating the most useful and interesting processes reported in the literature. The current reaction steps summaries in all Schemes for the domino sequences are efficient, but I would recommend including full catalytic cycles for some of them, such as those reported in Schemes 2B, 3B, 4A, 10A, 11E, 13B, 13C, 14. Visualizing these complex cycles will make the chemistry more accessible to the reader. The Outlook's emphasis on domino processes would be significantly strengthened by comparing one or two illustrated representative sequences

with a stepwise alternative reported in the literature. A side-by-side comparison of final yield for a representative product and possibly of some efficiency metrics would better demonstrate the superior performance of the domino approach.

Overall, the subject of this Outlook is of significant interest to a broad audience across both academic and industrial chemistry. It is well-organized and clearly written; therefore, I recommend it for publication once the suggested improvements and additions have been addressed.

Some corrections/typos are herein listed:

Page 5, in Scheme 2B under the arrow: “1-Carbonyl ene-type”

As general issue, please check authors’ surnames in the text and references section. Please correct as an example the following surnames: pag 15, second paragraph: Atiken versus Aitken; pag. 12, first paragraph: Latanzi versus Lattanzi.

Reviewer: 3

Comments to the Author

Tsogoeva and coworkers present an excellent outlook, which summarizes the evolution of various domino syntheses of complex heterocycles with a focus on organocatalytic, autoorganocatalytic and photo(redox) catalytic/mediated key steps both in nonenantioselective and enantioselective variants. This encompasses the developments of more than two decades and spans into the present, in particular, Tsogoeva‘s group has made several contributions, quite recently also on autocatalytic processes. The manuscript is very well written and definitely inspires the reader to „solve“ the synthetic puzzles with the given elementary steps of the domino syntheses in the schemes. The concept is nicely and systematically outlined in the beginning and clearly elaborated and illustrated in the following chapters. Overall, it represents a lead reference on recent developments of domino syntheses of complex heterocyclic scaffolds. Therefore, acceptance of this nice and instructive contribution is strongly recommended and should be accepted as is.

Author's Response to Peer Review Comments:

A point-by-point response to the concerns of reviewers:

Manuscript for ACS Central Science

Manuscript ID: oc-2026-005613

MS Type: Outlook

Title: **Domino Strategies in Heterocycle Synthesis: Advancing from Organocatalysis to Photo/Organo-Autocatalysis**

COMMENTS TO REVIEWERS:

We appreciate the Reviewers' efforts in carefully evaluating our manuscript and thank them for their constructive suggestions. We have considered all comments and made changes to the manuscript accordingly. All updates in the manuscript are highlighted. The following are the responses to the individual comments of the editorial office and the reviewers:

Editor's Comments:

- Graphics: If a figure has parts labeled (i.e. a, b, etc.), each part must be mentioned in the figure caption. Please see Schemes 2-12.

Response 1: Thank you for pointing this out. We have revised the captions of the Schemes to explicitly refer to and describe the individual labeled parts.

- Pull Quotes (Outlook): We encourage you to select 3 - 4 quotes from your Outlook that you would like highlighted in your paper. The quotes should be one sentencelong, unique to the Outlook and not from previously cited work. Please list your quotes at the end of the manuscript file.

Response 2: We have selected four pull quotes and listed them at the end of the manuscript, as requested. For ease of identification, the selected quotes have also been highlighted in green in the main text.

Reviewer: 1

Recommendation: Major revisions required.
Comments:
The outlook by Tsogoeva et all. describes domino strategies to access heterocyclic compounds with a particular focus on organocatalysis and photo-/organo-autocatalysis. The outlook is structured in 7 sections plus an outlook and a conclusion. The sections are well organized and selected examples are described within the evolution of domino synthesis.
The outlook deserves to be published after careful revisions of the following points.
Major revisions:
- In an attempt to be concise, the authors only depicted the general schemes of the reactions, summarising the sequence of the domino reactions. However, since domino reactions may have a complex mechanism, I would encourage the authors to reconsider the schemes and provide more information about the reaction mechanisms. For example, I would suggest:
Scheme 2A, add at least the structure of the acyl azolium intermediate
Scheme 3B, add the structure of the intermediate characterized by polarity inversion
Scheme 6B, add the structure of the reactive trienamine species
Scheme 6D, add the iminium-allenamine cascade, since it defined unprecedented
Scheme 7C, add the structure of the acyl azolium intermediate
Scheme 12, please add the structure of the excited state required for final cycloaddition

Response 3: We thank the Reviewer for the thoughtful review and the suggestions. We have added all key intermediates where space allowed: see Schemes 2A, 5A-C, 6A-E, 7A-C, 8A-B, 9A-C, 10A-B, 11A-E, and 12A. In addition, we presented selected catalytic cycles/reaction mechanisms: see new Figures 2-8.

Minor revisions:
-Page 2 line 53; please correct cinchona with Cinchona
-Page 5 line 45, right column, please correct 4-m ercapto-2-butenoate with 4-mercapto-2-butenoate
-Page 9 line 40, left column, please correct Nitrosobenzene with nitrosobenzene

Response 4: We thank the Reviewer for the comments. The suggested corrections have been implemented.

Page 16, right column, the first paragraph is in the wrong column.

Response 5: The paragraph is appropriately positioned at the end of chapter 7.2, as it provides a conclusion to the chapter. For a concise transition to this conclusive paragraph and to prevent an impression that it is in the wrong place, we added a sentence before the mentioned

paragraph: More broadly, this example underscores the synthetic potential of self-sustaining and autocatalytic processes. *Organo-autocatalysis*—a specific type of autocatalysis in which the catalyst is an organic molecule produced by the reaction itself—is emerging as a versatile platform for designing new highly sustainable reaction systems. …

-The text should be carefully revised to uniform formatting of word that should be written in italics like (N-; trig, tert-), like pages 5, 10 and 16 plus schemes 7, 10 and 11E
-Please specify the acronym DAR (scheme 13 and page 15), MBH page 7
-Scheme 4, please add the structure of carbobicycle
-Scheme 5, the font size of the catalysts is bigger than the other text; please correct
-Scheme 9A, please correct the structure of the product
-Please remove Figure 2, it does not add anything to the outlook

Response 6: We thank the Reviewer for the comments. The suggested revisions have been completed.

Reviewer: 2

Recommendation: Publish in ACS Central Science after minor revisions noted.

This Outlook highlights progress over time in enantioselective domino reactions, specifically focusing on heterocyclic and heteroaromatic compounds construction, their total synthesis, and self-catalyzed processes. By examining these transformations under organo- and photo-promoted conditions, this Outlook addresses a topic of significant relevance and interest to organic, medicinal, materials and industrial chemists. The manuscript, after a general introduction of the topic, has been sectioned starting from "Non enantioselective synthesis of heterocycles" then sectioned according to the mon-, multi and bridged- heterocycles and so on including stereoselective variants, illustrating the most useful and interesting processes reported in the literature. The current reaction steps summaries in all Schemes for the domino sequences are efficient, but I would recommend including full catalytic cycles for some of them, such as those reported in Schemes 2B, 3B, 4A, 10A, 11E, 13B, 13C, 14. Visualizing these complex cycles will make the chemistry more accessible to the reader.

Response 7: We thank the Reviewer for the positive feedback and the suggestions. We have added selected catalytic cycles: see new Figures 2-8. In addition, we presented key intermediates in Schemes 2A, 5A-C, 6A-E, 7A-C, 8A-B, 9A-C, 10A-B, 11A-E, 12A.

The Outlook's emphasis on domino processes would be significantly strengthened by comparing one or two illustrated representative sequences with a stepwise alternative reported in the literature. A side-by-side comparison of final yield for a representative product and possibly of some efficiency metrics would better demonstrate the superior performance of the domino approach.

Response 8: We thank the Reviewer for this suggestion and agree that a direct comparison between domino and stepwise approaches would be an effective way to highlight the advantages of domino processes. However, we were unable to incorporate such a comparison into the present Outlook. First, for the heterocyclic systems discussed herein, we could not identify suitable literature examples that would allow a meaningful and direct comparison between domino and corresponding stepwise syntheses of the same target structures. Second, the Outlook is already relatively comprehensive, and a thorough comparative analysis would require substantial additional discussion beyond the intended scope of the article.

Overall, the subject of this Outlook is of significant interest to a broad audience across both academic and industrial chemistry. It is well-organized and clearly written; therefore, I recommend it for publication once the suggested improvements and additions have been addressed. Some corrections/typos are herein listed: Page 5, in Scheme 2B under the arrow: "1-Carbonyl ene-type" As general issue, please check authors' surnames in the text and references section. Please correct as an example the following surnames: pag 15, second paragraph: Atiken versus Aitken; pag. 12, first paragraph: Latanzi versus Lattanzi.

Response 9: Thank you for these comments. The suggested corrections have been implemented

Reviewer: 3

Recommendation: Publish in ACS Central Science without change.

Comments:
Tsogoeva and coworkers present an excellent outlook, which summarizes the evolution of various domino syntheses of complex heterocycles with a focus on organocatalytic, autoorganocatalytic and photo(redox) catalytic/mediated key steps both in nonenantioselective and enantioselective variants. This encompasses the developments of more than two decades and spans into the present, in particular, Tsogoeva's group has made several contributions, quite recently also on autocatalytic processes. The manuscript is very well written and definitely inspires the reader to „solve" the synthetic puzzles with the given elementary steps of the domino syntheses in the schemes. The concept is nicely and systematically outlined in the beginning and clearly elaborated and illustrated in the following chapters. Overall, it represents a lead reference on recent developments of domino syntheses of complex heterocyclic scaffolds. Therefore, acceptance of this nice and instructive contribution is strongly recommended and should be accepted as is.

Response 10: We thank the Reviewer for the positive feedback.
